# Analysis of diverse double-strand break synapsis with Polλ reveals basis for unique substrate specificity in nonhomologous end-joining

**DOI:** 10.1038/s41467-022-31278-4

**Published:** 2022-07-01

**Authors:** Andrea M. Kaminski, Kishore K. Chiruvella, Dale A. Ramsden, Katarzyna Bebenek, Thomas A. Kunkel, Lars C. Pedersen

**Affiliations:** 1 Genome Integrity and Structural Biology Laboratory, National Institute of Environmental Health Sciences, National Institutes of Health, 111 TW Alexander Dr., Bldg. 101, Research Triangle Park, NC 27709 USA; 2 Department of Biochemistry and Biophysics, Lineberger Comprehensive Cancer Center, University of North Carolina at Chapel Hill, Chapel Hill, NC 27599 USA

**Keywords:** X-ray crystallography, DNA

## Abstract

DNA double-strand breaks (DSBs) threaten genomic stability, since their persistence can lead to loss of critical genetic information, chromosomal translocations or rearrangements, and cell death. DSBs can be repaired through the nonhomologous end-joining pathway (NHEJ), which processes and ligates DNA ends efficiently to prevent or minimize sequence loss. Polymerase λ (Polλ), one of the Family X polymerases, fills sequence gaps of DSB substrates with a strict specificity for a base-paired primer terminus. There is little information regarding Polλ’s approach to engaging such substrates. We used in vitro polymerization and cell-based NHEJ assays to explore the contributions of conserved loop regions toward DSB substrate specificity and utilization. In addition, we present multiple crystal structures of Polλ in synapsis with varying biologically relevant DSB end configurations, revealing how key structural features and hydrogen bonding networks work in concert to stabilize these tenuous, potentially cytotoxic DNA lesions during NHEJ.

## Abstract (web summary)

Using X-ray crystallography and nonhomologous end-joining assays, this study reveals structural features within Polλ that provide it with the ability to bridge and stabilize tenuous DNA double-strand break ends, allowing for religation.

## Introduction

Genomic integrity exists under imminent threat of breakage and damage due to endogenous exposures to reactive oxygen species generated by normal cellular metabolism, and from exogenous environmental exposures to ionizing radiation^1^. The most toxic form of genomic damage occurs when breaks in the phosphodiester backbone on opposing DNA strands cluster, forming DNA double-strand breaks (DSBs). Such breaks must be repaired quickly and efficiently, since their persistence within the human genome can lead to cancers and other diseases^2^. DSBs can be repaired through a variety of pathways, but nonhomologous end-joining (NHEJ) is favored in nonreplicating cells, or those that have yet to proceed through S phase of the cell cycle^3^. Though DSBs can lead to disastrous consequences, they can also prove beneficial when they occur in a controlled, programmed manner—through a specialized form of NHEJ known as V(D)J recombination, which is responsible for immunoglobulin gene maturation^4^.

**Fig. 1:**
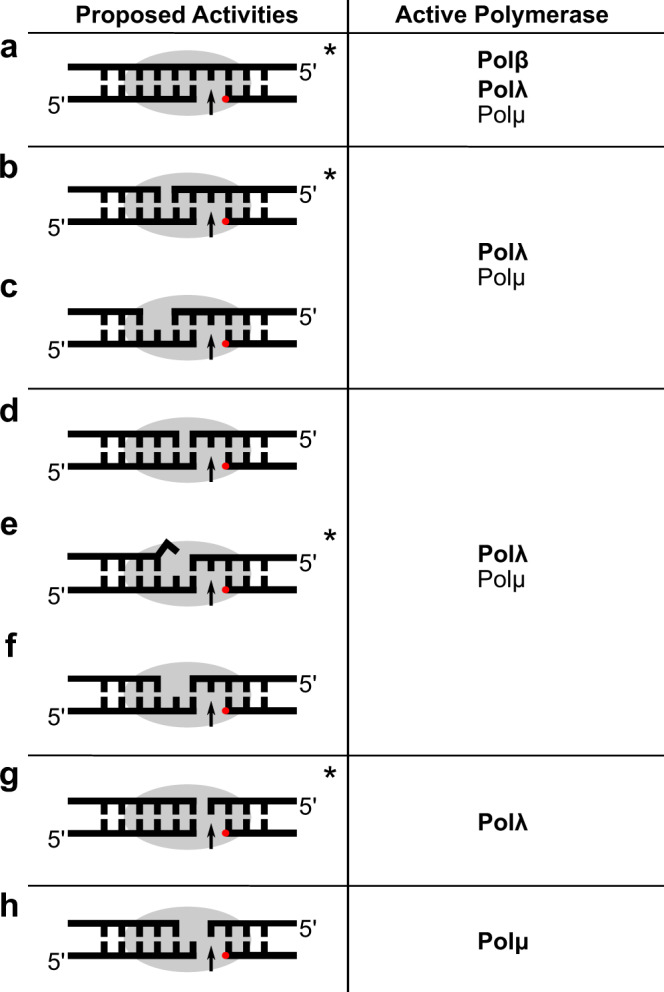
Schematic diagram of DNA substrates utilized by Family X polymerases. Single- and double-strand break DNA substrates are shown in stick on the left, with the rough footprint of the bound polymerase indicated by a gray oval. Locations of the 5ʹ-phosphate (if present) and the incoming nucleotide to fill the gap are given by a red dot and an upward black arrow, respectively. Polymerases that have been found to utilize each type of substate are listed on the right, with the identities of the polymerase shown to predominantly repair this configuration shown in bold. Types of substrates include single-strand breaks (**a**) and double-strand breaks with 2nt (**b**, **c**) or 1nt (**d**–**f**) complementarity, blunt-ended breaks (**g**), or noncomplementary breaks (**h**). Crystal structures of substrates with asterisks will be presented in this study.

NHEJ is carried out by a multiprotein complex capable of a variety of DNA sequence editing processes. In this pathway, broken DSB ends are bound by the Ku70/80 heterodimer, in conjunction with DNA-PK. Other processing factors are recruited to the complex (Artemis, DNA ligase IV, XRCC4, XLF, and DNA polymerases) to realign and trim the broken/damaged ends, fill in sequence gaps, and reseal the phosphodiester backbone^5^. The Family X polymerases (Pols), Polλ (gene name *Poll*), Polμ (gene name *Polm*), and terminal deoxynucleotidyl transferase (TdT), all participate in V(D)J recombination, each with a specific role^6–8^. Polλ and Polμ are widely expressed, template-dependent enzymes capable of repairing damage-induced DSBs in classical NHEJ, in addition to their roles in V(D)J recombination. Polμ has the unique ability to polymerize on entirely incompatible ends lacking any sequence homology at the break site, where the primer terminus is unpaired^9^ (Fig. 1). In contrast, Polλ is preferentially active on DNA substrates with a base-paired primer terminus. Polλ thus acts on DSB substrates containing 3′-overhangs with one or more complementary nucleotides that aid in repair of broken upstream/downstream ends—a context where Polμ is only modestly active. Polλ is also predominantly responsible for initiating synthesis from a blunt-ended upstream end, using as template the 3′-overhang from a downstream end in *trans*^10^. To better understand the structural determinants of how Polλ engages its preferred DSB substrates, we determined the X-ray crystal structures of its catalytic domain mediating end synapsis with a myriad of single- and double-strand break configurations that have been shown to be physiologically relevant substrates^9,10^ (Fig. 1). These structures demonstrate how Polλ engages DSB substrates, employing conserved hydrogen-bonding networks to synergistically stabilize both sides of the break simultaneously. A long, flexible loop region in the Polλ thumb subdomain was found to be specifically essential for blunt-end substrate utilization.

## Results

### Structural determinants of Polλ DSB substrate specificity

**Fig. 2:**
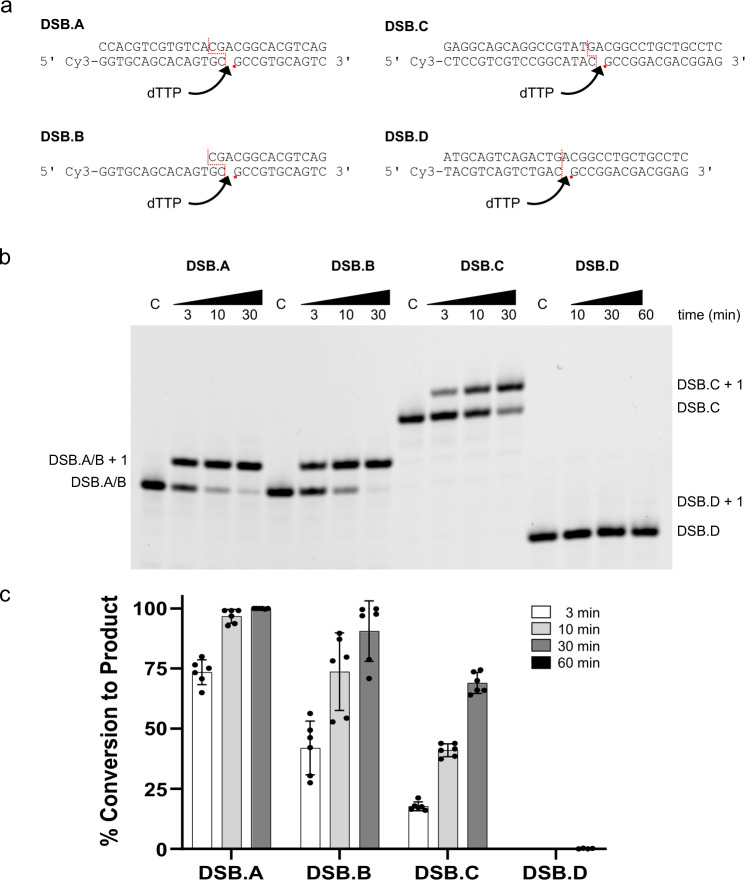
Polλ synapses and fills a variety of DSB end-configurations. **a** DSB substrates utilized for in vitro gap-filling assays. All substrates include a 5ʹ-Cy3-labeled upstream primer and a 5ʹ-phosphorylated (indicated by red dot) downstream primer. The break site location is indicated by a dashed red line. **b** Gap-filling activity of full-length wildtype Polλ on the different DSB end configurations examined in this study. **c** Quantifcation of gap-filling proficiency. The mean conversion from substrate to product is shown for each substrate, and the error bars correspond to the standard deviation. Reactions were prepared as biological replicates, with *n* = 6 for DSB substrates A–C, and *n* = 4 for DSB substrate D. The % conversion for the DSB.D substrate was less than 0.5% for all replicates, after a 60 min incubation.

In this study, we focused on how Polλ mediates synapsis on physiologically relevant DSB substrates containing either two nucleotides (2nt—DSB.A and DSB.B, similar to Fig. 1b, c), one nucleotide (1nt—DSB.C, similar to Fig. 1d–f), or no (blunt-end—DSB.D, similar to Fig. 1g) overlap between the upstream and downstream duplexes. We first employed a polymerization assay to determine how efficiently synapsis and subsequent gap-filling are accomplished on these substrates in vitro (Fig. 2a), in the absence of other NHEJ protein binding partners. These assays reveal that Polλ, alone, binds and fills gaps on substrates DSB.A, DSB.B, and DSB.C containing microhomology at the break site, which aids in bridging the upstream and downstream duplexes (Fig. 2b). The extent of microhomology also contributes to more efficient synapsis and gap-filling, as indicated by the increase in product formation in substrates containing two-nucleotide (DSB.A and DSB.B) rather than single-nucleotide microhomology (DSB.C). Without the aid of either NHEJ protein binding partners or sequence microhomology to bridge the upstream and downstream ends, Polλ struggles to efficiently fill the gap on the blunt-end DSB.D, as evidenced by the minimal amount of observed incorporation.

**Fig. 3:**
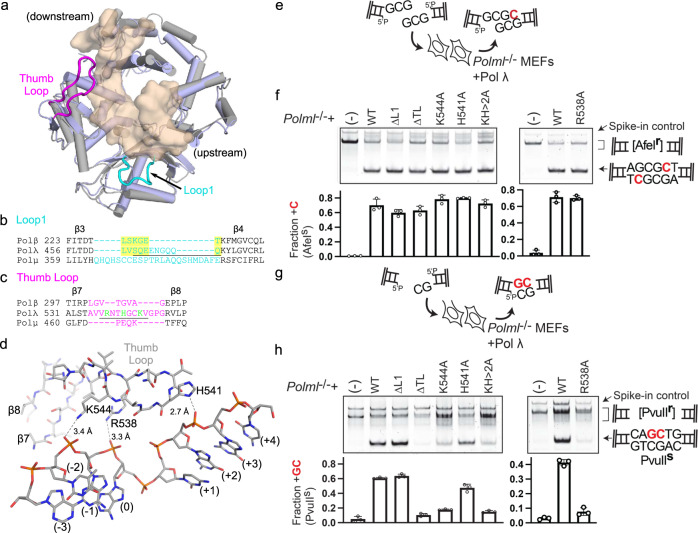
Determinants of DSB substrate specificity by Polλ. **a** Structural superposition of 1nt SSB crystal structures of Polλ (PDB ID code 1XSN^11^, protein in gray and DNA in transparent khaki surface) and Polμ (PDB ID code 4M04^12^, protein in light blue). Loop1 and the ‘thumb loop’ regions of Polλ are shown in cyan and magenta, respectively. **b** Structure-based sequence alignment of Loop1 (cyan) in Pols β, λ, and μ (structurally-equivalent regions highlighted in yellow). The Polλ ΔL1 variant was generated by deleting Ser463-Gln471 (underlined) and replacing them with Lys462-Thr465 from Polβ^13^. **c** Sequence alignment of ‘thumb loop’ (TL, magenta) of Pols β, λ and μ. The Polλ ΔTL variant was generated by deleting Val537-Val545 of Polλ (underlined) and fusing the ends of β-strands 7 and 8 with a single glycine residue. Thumb loop mutations used in this study are marked in green. **d** Interactions of the TL with an SSB substrate (PDB ID code 1XSN^11^, gray). Hydrogen bonds with the DNA template strand are shown as black dashed lines. **e** DNA fragments with partly complementary ends (GCG3ʹ overhangs) were introduced into *Polm*^*−/−*^*Poll*^*−/−*^ cells (*Polml*^*−/−*^). **f** Representative gel electrophoresis (top) identification of the fraction of NHEJ that is AfeI-sensitive and polymerase-dependent. Quantification of the average fraction of polymerase-dependent repair using GCG3ʹ DSB substrates from triplicate electroporations (bottom; error bars represent the standard deviation). **g** As in (**e**), except using noncomplementary blunt and GC3ʹ overhangs. **h** As in (**f**), except using PvuII sensitivity to identify polymerase-dependent repair.

Since repair of the blunt-end DSB substrates is primarily Polλ-specific^10^, we attempted to define the structural determinants bestowing Polλ, rather than Polμ, with this capability. The structural superposition of pre-catalytic SSB ternary complexes of these polymerases (Polλ PDB ID code 1XSN^11^ and Polμ PDB ID code 4M04^12^) reveal that their global structures are similar (RMSD of 1.55 Å over 257 Cα atoms), with differences primarily in the regions of Loop1 and the ‘thumb loop’ (Fig. 3a). Loop 1 influences polymerase fidelity in Polλ^13,14^, allows Polμ to repair DSB ends with unpaired primer termini^9^, and endows Polμ and TdT with template-independent synthesis behavior^15,16^. Loop1 varies in length between Family X members, with the longest loops observed in TdT or Polμ. A smaller loop exists in Polλ and dwindles to a mere turn in Polβ (Fig. 3b). However, Loop1 is disordered in all DNA-bound Polμ structures^12,17^. The sequence of Loop1 varies between the Family X members, but is well conserved between orthologs of Polλ^13^. The ‘thumb loop’ (TL), connecting β-strands 7 and 8 in the thumb subdomain, varies in length between the Family X polymerases, with the longest loop in Pol λ (Fig. 3c) exhibiting conservation of both length and sequence. The TL is considerably shorter in Polβ and exists as a turn in Polμ. This loop is often disordered in Polλ^18^ structures, but has been previously observed in an ordered conformation, juxtaposed with the template strand (PDB ID code 1XSN^11^, Fig. 3d). When ordered, residues in the TL (Lys544, Arg538, and His541) hydrogen bond with nonbridging oxygens along the template backbone. In order to determine whether Loop1 or the TL contribute to Polλ’s utilization of blunt-end DSB substrates during NHEJ, loop deletions or point mutations were created (Fig. 3b, c). Due to the relative inefficiency of synapsis and gap-filling on this substrate in vitro (Fig. 2b), a cell-based assay utilizing the endogenous cellular NHEJ machinery was instead used to explore the consequences of the loop deletions. All variants exhibited similar levels of end-joining on a 1nt gapped complementary DSB substrate with 2nt microhomology at the break site (Fig. 3e, f), suggesting these alterations do not broadly affect polymerase activity. The Polλ Loop1 variant^13^ (ΔL1) also exhibited end-joining similar to that of the wildtype on a blunt-end substrate with a 2nt 3ʹ-overhang (Fig. 3g, h). In contrast, the ΔTL deletion variant was markedly deficient in correct repair of this substrate, suggesting a significant role for the ‘thumb loop’ specifically, in blunt-end substrate utilization. To further dissect the role of the ‘thumb loop’, alanine substitution mutations of Arg538, Lys544 and His541 were also tested. The H541A mutant showed a subtle decrease in blunt-end DSB repair, while the R538A and K544A mutants were nearly as detrimental as the full loop deletion (Fig. 3h). Observation of such a substantial effect for R538A was surprising, given that Arg538 interacts with the template strand in the SSB, but the same interaction would not exist since this phosphate is not present in the blunt-end DSB. End-joining by the H541A/K544A double mutant was nearly indistinguishable from that of K544A, suggesting that the double mutations’ effects are not additive and that Lys544 plays the more significant role in extension from a blunt end. Interestingly, comparison of the in vitro and cell-based gap-filling assays show that, while Polλ struggles to independently synapse and repair the blunt-end DSB.D substrate in vitro (Fig. 2b), this difficulty appears to be entirely overcome by the presence of the NHEJ complex, as suggested by the similar amounts of complementary and blunt-end repair by the wildtype enzyme in vivo (Fig. 3f, h).

### Synapsis by Polλ on a fully complementary DSB substrate

**Fig. 4:**
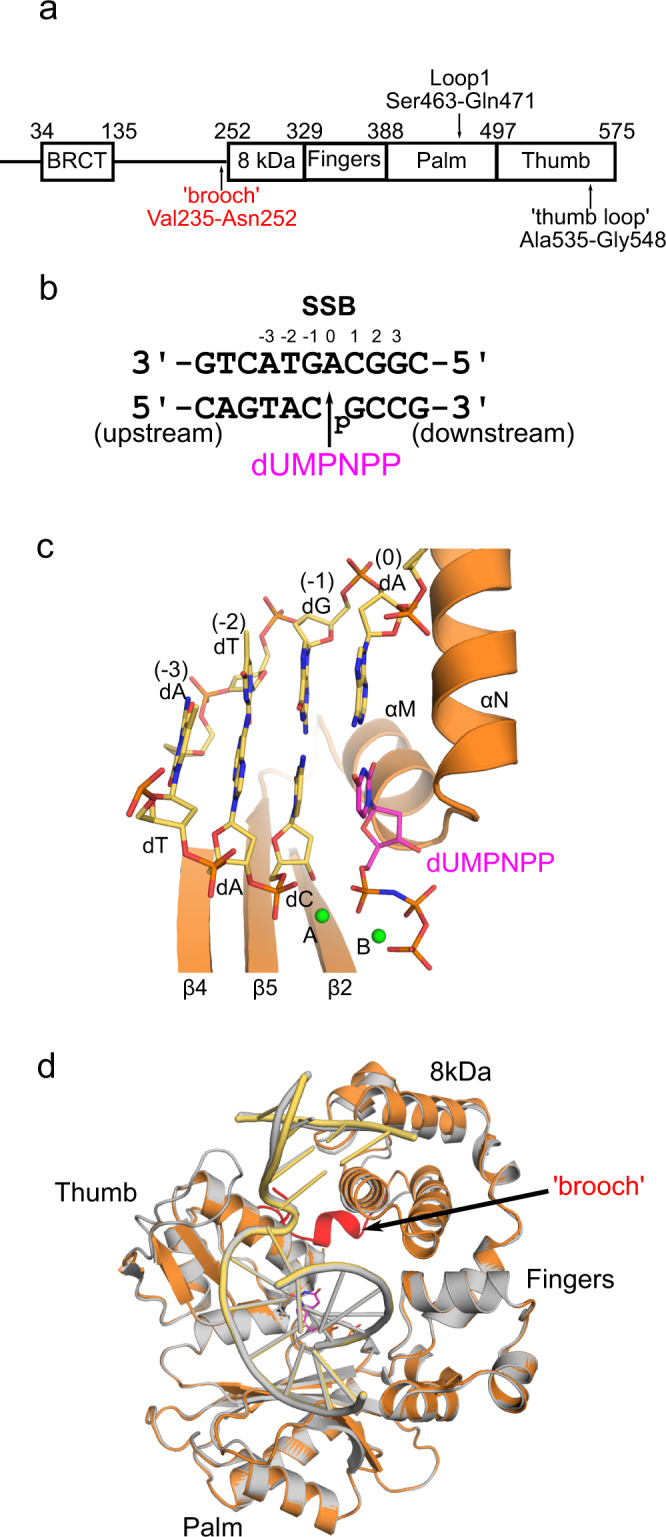
Characterization of Polλ ‘brooch’-containing constructs with a 1nt-gapped SSB. **a** Domain organization of human Polλ, with key structural features indicated. **b** 1nt-gapped SSB DNA substrate co-crystallized with the Polλ catalytic domain containing the ‘brooch’ motif. For the pre-catalytic complex, the template (chain T, top), upstream primer (chain P, bottom left), and 5′-phosphorylated downstream primer (chain D, bottom right) are pre-annealed and complexed with Polλ and an incoming nonhydrolyzable dUMPNPP nucleotide (magenta). **c** Zoomed-in view of the pre-catalytic SSB active site (protein in orange, DNA and incoming nucleotide colored as in (**b**), Mg^2+^ as green spheres). **d** Global superposition of the pre-catalytic ternary complexes of the Polλ catalytic domains in the absence (PDB ID code 2PFO^18^, gray) and presence (PDB ID code 7M07, protein in orange, DNA in pale yellow, dUMPNPP in magenta) of the ‘brooch’ motif’ (red).

Since Polλ was shown to be capable of synapsis and gap-filling in the absence of other NHEJ binding partners, we attempted to characterize the different synaptic complexes structurally, using X-ray crystallography. The Family X members involved in end-joining, Polλ, Polμ, and TdT, contain a small, conserved structural motif—known as the ‘brooch’—immediately preceding the 8 kDa subdomain (Fig. 4a). This motif facilitates end-bridging and gap-filling of DSB-containing DNA substrates, and has been hypothesized to aid in maintaining a closed conformation of the polymerase domain throughout the catalytic cycle^19^. Prior to the elucidation of the role of this motif, the ‘brooch’ historically had not been included in catalytic domain constructs used for crystallization, which were designed based on the structure of Polβ^11^. In order to increase the likelihood of crystallizing more tenuous, unstable DSB complexes, we utilized a ‘brooch’-containing catalytic domain construct of Polλ (Val235-Trp575) for the current study. This construct was first co-crystallized in a pre-catalytic ternary complex with a single-nucleotide (1nt) gapped single-strand break (SSB, Fig. 4b, similar to Fig. 1a) substrate (1.57 Å, PDB ID code 7M07, Supplementary Table 1, Fig. 4, Supplementary Fig. 1a). Superposition of this pre-catalytic complex with that of a previously reported Polλ^18^ pre-catalytic ternary complex lacking the brooch (PDB ID code 2PFO) shows that they are nearly indistinguishable (RMSD of 0.28 Å over 240 Cα, Fig. 4d and Supplementary Fig. 2), validating the use of the current SSB complex as a relevant reference model.

**Fig. 5:**
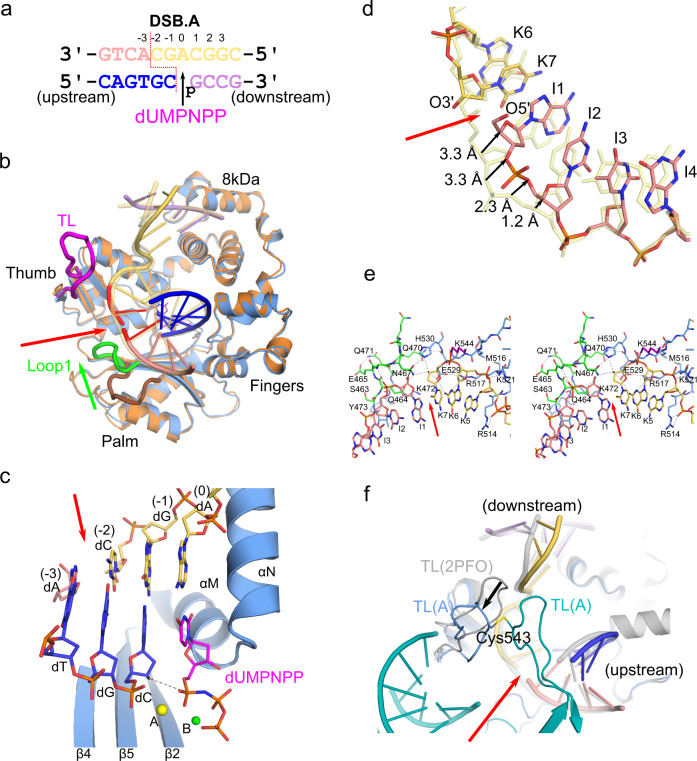
Polλ in complex with entirely complementary DSB.A. **a** Complementary DSB.A substrate (template strand broken between the −2 and −3 positions) co-crystallized with the Polλ catalytic domain (molecule B). The break site is indicated by a red dashed line. Upstream (left) template (pink, chain I) and primer (blue, chain J) are annealed separately from the downstream (right) template (pale orange, chain K) and primer (lavender, chain L) strands, and synapsis is mediated entirely by the polymerase. The DSB.A pre-catalytic quaternary complex is formed by addition of the nonhydrolyzable incoming dUMPNPP (magenta) nucleotide. **b** Global superposition of the pre-catalytic SSB ternary (orange) and complementary DSB.A quaternary (protein in blue, DNA and incoming dUMPNPP colored as in **a**) complexes. Positional differences of Loop1 (green), the thumb loop (TL, magenta), and the DNA template strand between the DSB.A and SSB structures are highlighted. Break site is indicated by a red arrow. **c** Structure of the DSB.A substrate bound in the Polλ active site (protein in light blue; DNA and incoming nucleotide drawn in stick and colored as in **a**). The Na^+^ and Mg^2+^ ions are shown as yellow and green spheres, respectively. The path between the primer terminal 3′-OH (axial) and the α-phosphate of the incoming dUMPNPP is shown (black dashed line). **d** Zoomed-in view of template strand shift in DSB.A (colored as in **a**), superimposed with the SSB (transparent khaki). **e** Stereo diagram of the interactions surrounding the break site (red arrow) in the DNA template strand (protein in light blue, DNA colored as in **a**), with hydrogen bonding interactions shown (black dashed lines). Residues from Loop1 and the ‘thumb loop’ are colored green and magenta, respectively. **f** Thumb loop in 7M0D (Ala535-Gly548) is ordered and differs in conformation from that observed in 2PFO^18^ (gray). Thumb loop conformation may be influenced by crystal packing interactions, as the thumb loop in molecule A (teal) interacts with the end of the upstream duplex from molecule B (colored as in **a**), and vice versa. The disulfide bond between Cys543 in each loop is indicated.

In order to understand how Polλ mediates synapsis of DSB ends during NHEJ, the ‘brooch’-containing catalytic domain construct was crystallized with a fully complementary DSB substrate containing a 3nt downstream template 3′-overhang, placing the break site between the −2 and −3 positions (DSB.A, Fig. 5a, similar to Fig. 1b). The DSB.A pre-catalytic complex (1.8 Å, PDB ID code 7M0D, Supplementary Table 1, Fig. 5b and Supplementary Fig. 3a) crystallized with two quaternary complexes per asymmetric unit, which display a high degree of structural similarity (RMSD of 0.21 Å over 272 Cα atoms, Supplementary Fig. 3b). The electron density for the active site in molecule B was slightly clearer (Supplementary Fig. 1b) than for molecule A and was therefore used for structural analysis. The active site of this pre-catalytic complex contains the nonhydrolyzable dUMPNPP bound in the nascent base pair binding site, along with two metals (Na^+^ in metal A site, Mg^2+^ in metal B site, Fig. 5c).

Superposition of the DSB.A and SSB complexes reveals global comparability (RMSD of 0.63 Å over 286 Cα atoms), though distinct differences are observed. Surprisingly, the template strand is shifted slightly from its position in the SSB complex (Figs. 5b and 5d). The templating base in the nascent base pair binding site (K7) and its immediate downstream neighbor (K6) occupy a very similar position, while the template strand upstream of the break site is rotated approximately 3 Å outward from its canonical position on the palm subdomain (Fig. 5d). The complementary primer strand nucleotides in this region maintain a similar position to those observed in the SSB complex, giving the appearance that the left-hand organization of the polymerase domain ‘holds’ tightly to the primer while allowing some movement of the template strand. The template strand shift coincides with a Loop1 (Ser463-Gln471, between β-strands 3 and 4, Fig. 5b, green) conformation different from those observed in either historical binary^20^/ternary^18,21^ Polλ complexes, or the SSB in the current study (Fig. 5b, brown). This new position allows Loop1 to interact with both the thumb subdomain (Gln470 NE2 – Glu529 O) and the DSB.A substrate, proximal to the break site (Fig. 5e and Supplementary Table 2). Rather than the backbone amide nitrogens from Gln471 and Lys472 forming a pocket anchoring the phosphate between template nucleotides at the −4 and −3 positions, this role is now played by the backbone amides of Glu465 and Glu466, with the amide of Ser463 contacting the next upstream phosphate (I3). Asn467 mediates a hydrogen-bonding network between Gln464 and His530. His530, Asn467, and Glu529 then work in concert to position the 3′-OH on residue K7 at the break site. Glu529 then extends the hydrogen bonding network to the downstream side of the break, with Arg517. Lys544 assumes an extended conformation and lies within hydrogen-bonding distance of the nonbridging phosphate oxygens between template nucleotides K6 and K7. Whereas Lys521 normally interacts with the phosphate backbone immediately downstream of the 90° bend in the template strand in the SSB (equivalent to the phosphate between K3 and K4), it now interacts with the phosphate immediately upstream of the bend (between K5 and K6).

In addition to alterations in Loop1, there is also a distinct difference in the order and conformation of the ‘thumb loop’ (TL) between β-strands 7 and 8 in the thumb subdomain (Fig. 5b, magenta). The ‘thumb loop’ is often disordered in Polλ structures, but is ordered in both molecules within the asymmetric unit of the DSB.A pre-catalytic quaternary complex and adopts the same conformation in each (Supplementary Fig 3b), which differs from that observed in other previously reported structures (PDB ID code 2PFO^18^, Fig. 5f, gray). Within the current lattice, the two ‘thumb loops’ are in close contact—mediated by a disulfide bond between the Cys543 sidechains—and interact with the DNA substrate.

### Polλ tolerates upstream DSB synaptic noncomplementarity

**Fig. 6:**
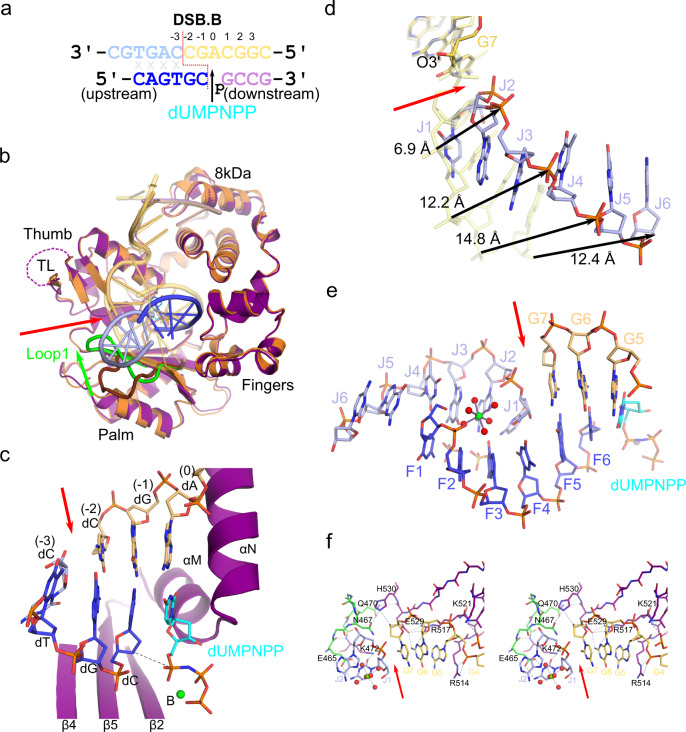
Polλ mediates synapsis via primer terminal pairing and accommodates noncanonical structures. **a** DSB.B substrate co-crystallized with Polλ catalytic domain (molecule A), with break site indicated by a red dashed line. Upstream (left) primer (blue, chain F) was annealed separately from the downstream (right) template (pale orange, chain G) and primer (lavender, chain H) strands, and synapsis is mediated by the polymerase. Surrogate of upstream template (light blue, chain J) is the 5′-end of the upstream primer from a neighboring molecule, which mispairs (gray x) with the current upstream primer (blue). DSB.B pre-catalytic quaternary complex is formed by addition of nonhydrolyzable incoming dUMPNPP (cyan) nucleotide. **b** Global superposition of pre-catalytic SSB ternary (protein in orange, DNA in transparent yellow) and DSB.B synaptic (protein in purple, DNA and incoming dUMPNPP colored as in **a**) complexes. Positional differences of Loop1 (green) and the DNA template strand between the DSB.B and SSB structures are highlighted. Break site is indicated by a red arrow. **c**. Structure of the DSB.B synaptic complex bound in the Polλ active site (protein in purple; DNA and incoming nucleotide colored as in **a**). While the metal A site is unoccupied, while the metal B site is fully occupied by a Mg^2+^ ion (green sphere). The path between the primer terminal 3′-OH (axial) and the α-phosphate of the incoming dUMPNPP is shown (black dashed line). **d** Zoomed-in view of the template strand shift in DSB.B (colored as in **a**), superimposed with the template strand from the SSB (transparent khaki). **e** The upstream duplex (primer in blue, upstream template from neighboring molecule in light blue) forms an entirely mispaired duplex, mediated by hydrogen bonding interactions with a hydrated Mg^2+^ ion (green, with coordinating waters, red spheres) and alternate conformations of sulfate ions (yellow) from the mother liquor. Expanded views of individual mispairs are displayed in Supplementary Fig. 5. **f** Stereo diagram of interactions surrounding the break site (red arrow) in the DNA template strand (protein in dark purple, DNA colored as in **a**), with hydrogen bonding interactions drawn as black dashed lines. Residues from Loop1 are shown in green.

We next attempted to co-crystallize the Polλ catalytic domain in complex with a substrate similar to DSB.A, but with a single-nucleotide template gap at the −3 position (similar to Fig. 1c). However, the resulting crystals exhibited an entirely unexpected—yet fortuitous—synaptic arrangement (DSB.B, Fig. 6a) in *trans*, where the upstream template oligo never annealed. These crystals were subsequently reproduced in the absence of the upstream template oligo (2.25 Å, PDB ID code 7M0E, Supplementary Table 1 and Fig. 6), wherein the 5′-end of the upstream primer forms an entirely noncomplementary, mispaired duplex with the 5′-end of the same primer from a neighboring molecule in the asymmetric unit (Supplementary Fig. 4). The pre-catalytic complex was trapped by binding of the nonhydrolyzable dUMPNPP (Fig. 6b, c). Despite vastly different crystal packing, the DSB.B synaptic complex displays a high degree of global structural comparability to that of the SSB complex (RMSD of 0.53 Å over 281 Cα atoms, Fig. 6b). Components within the active site (primer terminus and correct base pairs at the 0, −1, and −2) are observed in their expected positions (Fig. 6c), however, the farther upstream along the DSB.B substrate from the catalytic center, the more this synaptic complex deviates from canonical geometry (Fig. 6d). For the upstream primer, a high degree of structural similarity is maintained while the substrate lies within the footprint of the palm subdomain (nucleotides F4-6), likely anchored by a myriad of hydrogen bonding interactions with the palm subdomain and coordination of the Na^+^ ion in the Helix-hairpin-Helix (HhH) 2 site. The upstream primer progressively diverges from the canonical primer strand trajectory (distances of 1.1 Å, 2.4 Å, and 4.4 Å at nucleotides F3, F2, and F1, respectively). This deviation is likely due to mispairing of the upstream primer strand from the neighboring molecule, which serves as a surrogate for the missing upstream template strand. This surrogate template strand no longer occupies the conventional position of the template strand, as observed for the SSB or DSB.A complexes and has drastically shifted—by 6.9 Å at the break site to 14.8 Å closer to the upstream end (Fig. 6d). The noncomplementary interactions of the 5′-ends of these oligos creates an entirely mispaired region upstream of the break site (Fig. 6e and Supplementary Fig. 5), juxtaposed between the coordination of a solvent-exposed, hydrated Mg^2+^ ion and partially ordered sulfate ions from the mother liquor.

Interactions downstream of the break site are largely conserved in the DSB.B synapse, similar to those observed in the DSB.A complex. Arg514 stacks with the templating base and anchors the phosphate backbone at the 90 ° bend between downstream template nucleotides G4-5 and Lys521 lies within hydrogen-bonding distance of the next phosphate downstream (Fig. 6f and Supplementary Table 2). Arg517 extends into the minor groove and interacts with the G6 base and the sidechain of Glu529, which participates in a small hydrogen bonding network with the G6 base and the 5′-OH of G7. His530 also helps to position the 5′-OH and mediates interactions with Asn467. Because the surrogate template residue J1 is rotated inward into the duplex, rather than maintaining the continuous flow of the phosphate backbone within the binding cleft, its base now participates in multiple hydrogen-bonding interactions, with the sidechains Lys472 and Glu465. Loop1 adopts a conformation different from either of those observed in the SSB and DSB.A complex (Fig. 6b, green), which cradles the unconventional DNA substrate configuration upstream of the break site.

### Polλ can accommodate mispairing near the DSB break site

**Fig. 7:**
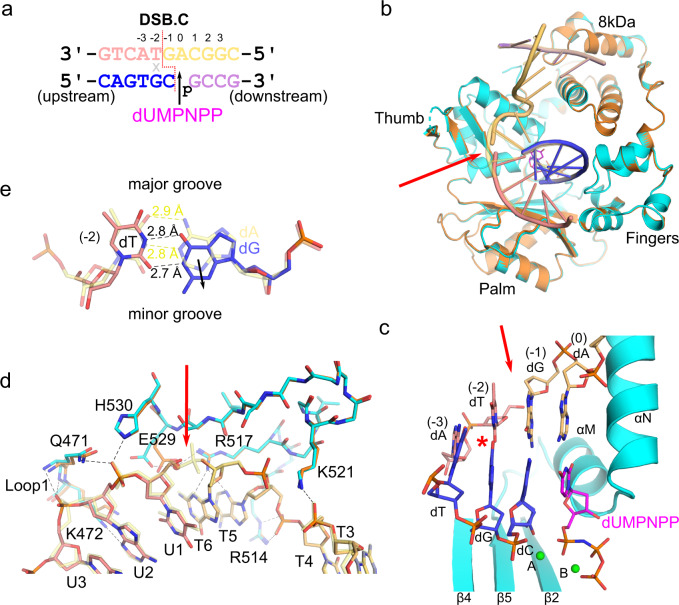
Polλ can accommodate mispairs proximal to the break site. **a** DSB.C substrate, with template strand broken between the −1 and −2 positions, co-crystallized with the Polλ catalytic domain. This substrate also contains a G:T mispair (indicated by gray x) immediately upstream of the break site (red dashed line), at the −2 position. Upstream (left) template (pink, chain U) and primer (blue, chain P) are annealed separately from the downstream (right) template (pale orange, chain T) and primer (lavender, chain D) strands, and synapsis is mediated by the polymerase. The DSB.C pre-catalytic quaternary complex is formed by the addition of the nonhydrolyzable incoming dUMPNPP (magenta) nucleotide. **b** Global superposition of the pre-catalytic SSB ternary (orange) and DSB.C quaternary (protein in cyan, DNA and incoming dUMPNPP colored as in **a**) complexes. **c** Structure of the mispaired (red asterisk) pre-catalytic DSB.C substrate bound in the Polλ active site. Mg^2+^ ions are shown as green spheres. **d** Superposition of the pre-catalytic SSB (protein in orange, DNA template in transparent pale yellow) and mispaired DSB.C (protein in cyan, DNA colored as in **a**, water molecule as red sphere) complex for comparison of interactions surrounding the DSB break site (red arrow). Hydrogen bonding interactions with the mispaired DSB.C are drawn (black dashed lines). **e** Superposition of the base pair at the −2 position in either the SSB (correct base pair, transparent yellow) or mispaired DSB (template dT:primer dG in pink and blue, respectively), with measured hydrogen bonding distances drawn as yellow (correct) or black (incorrect) dashed lines. Solid black arrow indicates subtle shift of the mispaired primer dG toward the minor groove.

To further explore Polλ’s requirement for a paired primer terminus in gap-filling during NHEJ^9,22^, we next co-crystallized its catalytic domain in complex with a DSB substrate comprised of an upstream duplex and a 2nt downstream template 3′-overhang, yielding a break between the −1 and −2 positions (DSB.C, Fig. 7a, similar to Fig. 1e). Therefore, the polymerase may mediate DSB synapsis of the upstream and downstream duplexes, aided by a single correct base pair on the primer terminus. This substrate also contains a G:T (primer:template) mismatch immediately upstream of the break site, which could give insight into Polλ’s tolerance for such nonhomology in DSB substrates. The pre-catalytic quaternary DSB.C complex was assembled in a similar fashion to that of DSB.A-B but crystallized with the same space group and unit cell dimensions (2 Å, PDB ID code 7M0B, Supplementary Table 1 and Fig. 7) as the 1nt SSB. Structural superposition shows a high degree of similarity between the DSB.C pre-catalytic complex and that of the SSB (RMSD of 0.224 Å over 302 Cα atoms) (Fig. 7b). There is clear density for the backbone discontinuity between the −1 and −2 positions (Supplementary Fig. 1d). The DNA substrate in the DSB.C complex superimposes well with that of the SSB—therefore, interactions with the substrates are largely identical between these complexes (Fig. 7d and Supplementary Table 2). This observation suggests that Polλ likely utilizes comparable DNA substrate binding modes, whether the template strand is unbroken or contains a break within one or two positions upstream from the nascent base pair binding site (0 position). There are a few subtle differences observed between these complexes. Lys472 exhibits conformational flexibility, which often places the sidechain within hydrogen-bonding distance of template bases upstream of the break site. Glu529 is observed in distinct rotamers in each structure, which allow it to form different interactions in each case. In the current conformation, a water molecule mediates a hydrogen bond bridge between Glu529 and Arg517. The flexibility of this residue is intriguing, since previous studies have shown that Glu529 functions as a sensor preventing mutagenic bypass of a template strand 8-oxoG^23^.

The presence of the G:T mispair near the primer terminus does not overtly distort the upstream duplex, though the mispaired primer dG, is shifted very slightly (~1 Å) toward the minor groove in a wobble conformation (Fig. 7e). The incorrect G:T mispair allows for two hydrogen bonds between the bases, which are of similar distance (2.8 and 2.7 Å) and geometry to those in the correct A:T base pair in the SSB complex (2.9 and 2.8 Å). Both the primer dG and the template dT bases are observed in the *anti* conformation, which maintains the cognate base shape^24^ of a purine:pyrimidine base pair. This suggests that Polλ would likely be tolerant of similar minimally-distorting mismatches proximal to DSB break sites.

### DSB synapsis by Polλ on a blunt-ended DSB substrate

**Fig. 8:**
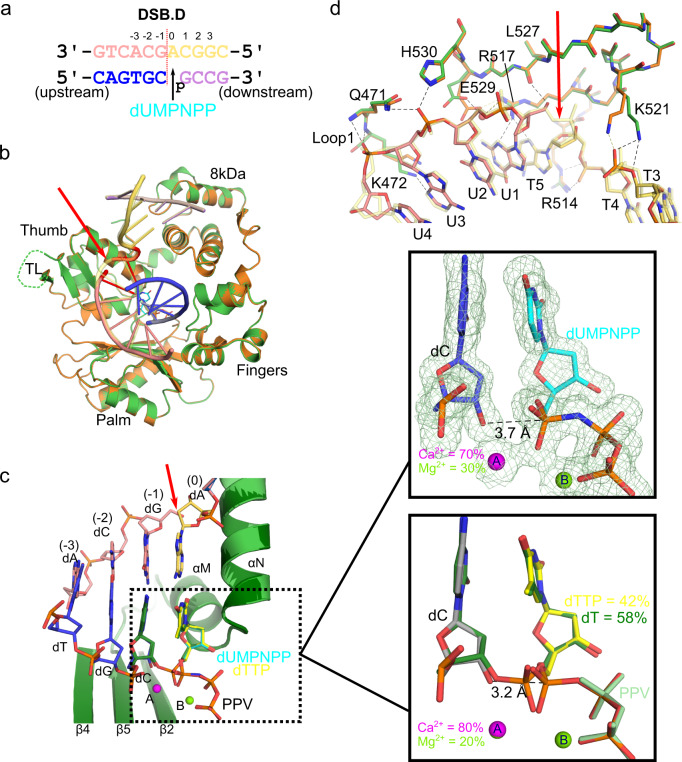
Polλ can address blunt-ended DSBs and SSBs in a similar manner. **a** Blunt-ended DSB substrate (DSB.D) co-crystallized with the Polλ catalytic domain. The break site is indicated (red dashed line). Upstream (left) template and primer (pink and blue, respectively) are annealed separately from the downstream (right) template (pale orange) and primer (lavender) strands, and synapsis is mediated entirely by the polymerase. The DSB.D pre-catalytic quaternary complex is formed by addition of the nonhydrolyzable incoming dUMPNPP (cyan) nucleotide. **b** Global superposition of the pre-catalytic SSB ternary (orange) and blunt-ended DSB quaternary (protein in green, DNA and incoming dUMPNPP colored as in **a**) complexes. **c** Structure of the DSB.D substrate bound in the Polλ active site, with the pre- (protein in light green, DNA and incoming nucleotide colored as in **a**) and partially incorporated (protein and DNA in dark green, unincorporated hydrolyzable incoming dTTP nucleotide in yellow, PDB ID code 7M0A) substrates, with zoomed-in views of each complex included to the right (top, pre-catalytic, with 2*F*_*o*_*-F*_*c*_ electron density contoured to 1σ; bottom, partially incorporated, with unincorporated primer terminus and dTTP in gray and yellow, respectively). The Ca^2+^ and Mg^2+^ ions are shown as magenta and green spheres, respectively. Though the metal in the B site is modeled at full occupancy, based on geometry and coordination distances, a mixture of metals is observed in the A site. Occupancies for the metal A site mixture and newly incorporated nucleotide are indicated in each case. **d** Superposition of the pre-catalytic SSB (protein residues in orange, DNA template in transparent pale yellow) and blunt-ended DSB.D substrates (protein in green, DNA template strands colored as in **a**, water molecule as red sphere) for comparison of interactions surrounding the DSB break site (red arrow). Hydrogen bonding interactions with the DSB or the SSB are drawn as dashed lines (black or yellow, respectively).

We then assessed how Polλ mediates end synapsis of a DSB substrate with a blunt-ended upstream duplex and a single nucleotide 3′-overhang from the downstream duplex (DSB.D, Fig. 8a, similar to Fig. 1g), as previous studies have shown that repair of this substrate configuration is primarily Polλ-dependent^10^. Since these quaternary complex crystals are grown in a mother liquor containing a high concentration of Ca^2+^, which is incompatible with nucleotide incorporation, we attempted to exchange the active site metals with Mg^2+^ (pre-catalytic complex, 1.65 Å, PDB ID code 7M09, Supplementary Table 1), and then to exchange the nonhydrolyzable dUMPNPP with hydrolyzable dTTP to allow for *in crystallo* nucleotide incorporation (partially incorporated complex, 1.83 Å, PDB ID code 7M0A, Supplementary Table 1 and Fig. 8c). The structural superposition of the DSB.D pre-catalytic complex shows that it is nearly indistinguishable from the pre-catalytic SSB-bound complex (RMSD of 0.11 Å over 292 Cα atoms, Fig.8b). There is a gap in the electron density between the templating base (0 position) and the −1 position (Supplementary Figs. 1e, inset and 1f). In the pre-catalytic complex, a mixture of metals is observed in the A site (refined with occupancies of 70% and 30% for Ca^2+^ and Mg^2+^ ions, respectively), indicating incomplete metal exchange at that location. Full occupancy for Mg^2+^ is observed in the metal B site. The primer terminus is observed in a conformation intermediate between C2′-*endo* and C3′-*endo*, placing its 3′-OH neither fully axial nor fully equatorial (distance of 3.7 Å to α-phosphate of dUMPNPP, Fig. 8c, upper inset). Interestingly, the electron density for this intermediate conformation is very clear and distinct, rather than exhibiting an elongated or diffuse appearance usually indicative of a mixture of axial and equatorial conformations. Exchange of the dUMPNPP for dTTP yielded a mixture of incorporated dTMP (58%) and unincorporated dTTP (42%) (Supplementary Fig. 1f). Similar to the pre-catalytic complex, a mixture of ions is again observed in the metal A site (80% and 20% occupancy for Ca^2+^ to Mg^2+^, Fig. 8c, lower inset). Interestingly, the 3′-OH on the unincorporated primer terminus adopts the fully equatorial position, 3.2 Å from the α-phosphate of the dTTP. The pyrophosphate leaving group (PPV) is still present and ordered in the active site.

The template strands in the DSB.D and SSB complexes superimpose well and therefore participate in largely similar interactions (Fig. 8d, Supplementary Table 2). Interactions common to both structures include a stacking interaction of the Arg514 sidechain with the templating base, as well as two putative hydrogen bonds between the sidechain and the nonbridging phosphate oxygens between nucleotides T4 and T5 (Supplementary Table 2). Arg517 extends along the minor groove and makes two sequence-nonspecific hydrogen bonds with the T5 and U1 bases, on either side of the break. The His530 and Gln471 sidechains each hydrogen bond with a nonbridging phosphate oxygen between upstream template U2 and U3. Backbone amides from Gln471 and Lys472 anchor the phosphate backbone between U3 and U4. There are a few subtle differences observed between the DSB.D and SSB complexes (Supplementary Table 2). In the DSB.D complex, the 5′-OH on the upstream template rotates outward toward solvent, rather than inward toward the protein, and makes a putative hydrogen bond with the backbone carbonyl of Leu527. The Glu529 sidechain makes a hydrogen bond with the Arg517 sidechain in the SSB complex but adopts a different conformation in the DSB.D complex, instead utilizing a water molecule to bridge the interaction with Arg517 (as observed in the complex with DSB.C). Lysine residues 472 and 521 assume different conformations in each complex. In the SSB, Lys521 interacts with the nonbridging phosphate oxygen between T3 and T4. In the DSB.D complex, this lysine sidechain is shifted slightly farther downstream and could hydrogen bond with either the T3-T4 nonbridging or bridging oxygens. Lys472 in the DSB.D lies within hydrogen-bonding distance of the U3 base but assumes a different conformation in the SSB-bound complex, where this interaction is not observed. Interestingly, the ‘thumb loop’ region is disordered in this structure, as it is in that of the SSB (Fig. 8b, green dashed line). It is possible that the ‘thumb loop’ is required for interactions stabilizing the initial, tenuous steps of end-bridging, and is no longer required once a stable complex has been formed (consistent with the steady-state conditions within a crystal lattice).

## Discussion

**Fig. 9:**
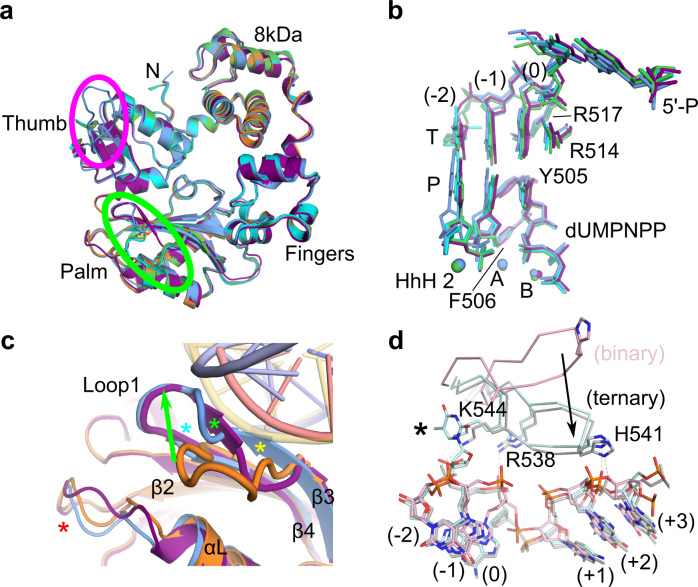
Loop1 and ‘thumb loop’ adopt varying conformations to cradle different substrate configurations. Superpositions of the global protein (**a**) and active site components (**b**) of the pre-catalytic SSB (orange), complementary DSB.A (blue) and DSB.B (purple), mispaired DSB.C (cyan), and blunt-ended DSB.D (green) synaptic complexes. **c** The upstream template region of the DNA substrate adopts variable trajectories in different complexes, which are, in turn, cradled by varying Loop1 conformations (SSB, protein in orange, DNA in transparent yellow; DSB.A, protein in blue, DNA in pink; DSB.B, protein in purple, DNA in light blue). These conformations alter the length and configuration of β-strands 3 (yellow asterisk) and 4 (cyan asterisk) and intervening secondary structure (green asterisk), which likely influence the position of the loop between β-strand 2 and α-helix L (red asterisk). **d** Structural superpositions of the Polλ ‘thumb loop’ observed in an ordered conformation in the canonical 1nt SSB binary (PDB ID code 1XSL^11^, pink) and 1nt SSB pre-catalytic ternary (PDB ID code 1XSN^11^, gray) complexes, relative to the 1nt strand-slippage intermediate (PDB ID code 2BCV^25^, light cyan, with extrahelical base marked with a black asterisk). Putative hydrogen bonding interactions are indicated by dashed lines in colors corresponding to the structure. Movement of the ‘thumb loop’ upon nucleotide binding is shown by a black arrow.

Analysis of the SSB and DSB crystal structures presented in this study allows for a breakdown of the Polλ active center into its most critical components. Regardless of the substrate configuration, the protein subdomains, DNA substrates, and active site residues assume the canonical ‘active/closed’ conformation (Fig. 9a, b). In each case, the 5′-phosphate on the downstream duplex lies within the same pocket on the surface of the 8 kDa domain, likely aiding in binding and orientation of the downstream end of the gap. 5′-phosphate binding, juxtaposed with the 90° bend in the template phosphodiester backbone immediately upstream of the +1 position in the downstream duplex, properly positions the template base (0 position) in the nascent base pair binding site, stacked by Arg514. Arg517 extends into the minor groove, and Tyr505/Phe506 have shifted from their ‘inactive/open’ to their ‘active/closed’ conformations observed upon nucleotide binding^11^. Coordination of the Na^+^ ion in the HhH2 site and an intricate network of hydrogen bonding interactions anchor the upstream duplex to the palm subdomain through the primer strand. These interactions, aided by base stacking and pairing at the −1 position, correctly align the primer terminus for catalysis. The structural similarity of these components, despite varying end configurations and potential crystal packing influences, suggest that they are essential factors for synapsis by Polλ.

Detailed comparison of interactions between the Polλ catalytic domain and the various configurations of DSB substrates used in this study reveal that the hydrogen bonding networks downstream of the break are largely conserved (Figs. 5e, 6f, 7d, 8d and Supplementary Table 2), regardless of end configuration. However, interactions proximal to and upstream of the break site vary as the location of the template strand break moves farther from the nascent base pair binding site. Though previous studies have established that Polλ participates in repair of DSB substrates with a minimum of single nucleotide base pairing at the primer terminus^9,22^, substrates with two or more base pair microhomology are also commonly used for in vitro and in vivo NHEJ assays^10,19^. Synaptic stability likely increases with increasing microhomology length, a supposition supported by the observation that Polλ generates more product using DSB substrates with 2nt rather than 1nt overlap (Fig. 2b, c). Comparison of the DSB.C crystal structure presented in this study with the recently reported complex of Polμ bound to a similar end configuration (template strand discontinuity between the −1 and −2 positions, PDB ID code 6WIC^17^) shows a high degree of similarity between the two proteins in the vicinity of the template nucleotide opposite the primer terminus. A hydrogen bond exists between the protein and the base at that position in Polλ (Arg517 NH1—T6 N3) and a similar interaction is conserved in Polμ (Arg445 NH1—T6 O2). Another hydrogen bond between Arg517 NH1 and the O4ʹ of the ribose sugar is possible, which would be considered a long-distance interaction in Polμ. Therefore, the means by which Polλ directly ‘senses’ the primer terminal base pair, and the structural underpinnings of its absolute requirement for this base pair, remains unclear.

Global superpositions of the pre-catalytic SSB and DSB synaptic complexes show that the Polλ catalytic domain adopts similar protein conformations, regardless of the DSB end configuration presented (Fig. 9a). The most notable structural differences are observed in Loop1 and the ‘thumb loop’—alternations that could serve to stabilize a multitude of structurally diverse DSB end configurations (Fig. 9a, green and magenta circles, respectively). In the DSB.A complex, the upstream template strand is rotated slightly outward from the palm subdomain and there is a concomitant shift of Loop1. This shift lengthens both β-strands 3 and 4, which would create a steric clash with the template strand in the conventional location (Fig. 9c, yellow and cyan asterisks, respectively). In the DSB.B complex, Loop1 adopts a largely similar conformation to that observed in DSB.A, with the extension of β-strand 4. In DSB.B, however, there is a deviation from canonical geometry for β-strand 3 in the vicinity of Gln464-Glu465, which allows this region to interact with bases in the mispaired upstream duplex (Fig. 9c, green asterisk). Though the conformations of Loop1 in the DSB.A and DSB.B complexes are largely similar in trajectory, each would create a steric clash with the template strand of the other complex (Fig. 9c). The structures presented in the current study therefore provide structural evidence that Polλ’s Loop1 may play a similar stabilizing role for the upstream template region of DSB substrates, as has been hypothesized for the analogous loop in Polμ^16,17^.

The ‘thumb loop’ is a structural feature unique to Polλ, compared to the other Family X polymerases, and is often disordered in crystal structures or displays substantial thermal motion when present. In the current study, the ‘thumb loop’ is ordered only in the DSB.A complex and makes contacts only with DNA template strands from other molecules in the asymmetric unit (Fig. 5f). In a similar fashion to that of Loop1, the ‘thumb loop’ has previously been shown to adopt different conformations in binary and pre-catalytic ternary SSB complexes (PDB ID codes 1XSL and 1XSN, respectively^11^), with the loop moving to cradle the template strand upstream and downstream of the bend in the DNA simultaneously (Fig. 9d). In the ordered conformation proximal to the DNA, residues in the loop (Arg538, His541, and Lys544) lie within hydrogen-bonding distance of the phosphate backbone. Additionally, crystal structures of Polλ in complex with a strand-slippage intermediate containing an extrahelical base between the −1 and −2 positions (PDB ID code 2BCV^25^) display a similar conformation of the ‘thumb loop’. However, in this structure, Lys544 from the ‘thumb loop’ employs Van der Waals forces to interact with the planar face of the extruded base. Alanine substitution of Lys544 markedly decreases activity on blunt-end DSB.D, with no deleterious effects on utilization of the complementary DSB with 2nt microhomology at the break site (Fig. 3e–h). In contrast, mutation of His541 has only minimal effect on usage of the blunt-end substrate. These results suggest that the limiting step of blunt-end DSB synapsis may lie with the difficulty of correctly positioning the upstream duplex and is strongly dependent on the presence of ‘thumb loop’ interactions for assembly.

In-depth structural comparisons of the variety of Polλ synaptic complexes, such as the ones presented in this study, provide invaluable insight into how this polymerase engages its DSB substrates and mediates end bridging in preparation for gap-filling during NHEJ. Such structural information presents a firm foundation for future understanding of DSB synapsis within the broader context of the full NHEJ complex.

## Methods

### Expression and purification of Polλ proteins

Full-length (Met1-Trp575) or catalytic domain constructs (Val235-Trp575) of human DNA Polλ were cloned into the NotI/EcoRI restriction sites of the pGEXM vector^12^. Constructs were transformed into Rosetta2 (DE3) cells and expressed in large-scale in LB medium supplemented with 100 μg/mL ampicillin and 35 μg/mL chloramphenicol. Once the cultures reached an OD_600nm_ of 0.8, the temperature was reduced to 18 °C for 30 min, when protein expression was induced by addition of isopropyl-β-D-thiogalactoside (IPTG) to a final concentration of 0.4 mM, and continued overnight at 18 °C. The cells were pelleted by centrifugation and lysed by sonication in 25 mM Tris pH 8, 750 mM NaCl, 5% glycerol, 1 mM DTT. Polyethyleneimine was added to a final concentration of 0.1% (v/v), dropwise, while stirring at 4 °C for 5 min. The lysate was subsequently clarified by centrifugation, bound in-batch to glutathione sepharose 4B resin (Cytiva), and subjected to on-resin TEV protease cleavage in 25 mM Tris pH 8, 500 mM NaCl, 5% glycerol, 1 mM DTT overnight at 4 °C. Polλ proteins were further purified by size exclusion chromatography, dialyzed to storage buffer containing 25 mM Tris pH 8, 100 mM NaCl, 5% glycerol, 1 mM DTT and concentrated to 10–15 mg/mL. Concentrated proteins were flash frozen in liquid nitrogen, and stored at −80 °C.

### Generation of full-length Polλ mutants

Substitution mutants and deletion variants (ΔL1 with Loop1 residues Ser463-Gln471 deleted and replaced with KGET loop sequence from Pol β^13^; ΔTL, deletion of thumb loop residues Val537-Val545 and insertion of a single glycine residue to bridge the gap) were generated in the full-length human Polλ using QuikChange mutagenesis (Agilent). All mutant clones were fully sequenced by Genewiz. Wildtype, mutant or variant proteins were expressed from Rosetta2 (DE3) cells in Terrific broth (supplemented with 100 μg/mL ampicillin and 35 μg/mL chloramphenicol) to increase yield in small scale. All mutants behaved similarly to wild-type protein during size exclusion chromatography and are therefore likely to be properly folded. The final storage buffer contained 25 mM Tris pH 8, 200 mM NaCl, 5% (v/v) glycerol, and 1 mM DTT.

### Analysis of in vitro DSB gap-filling by Polλ

The final reaction mixture (30 µl) contained 50 mM Tris pH, 7.5-8, 1 mM DTT, 4% (v/v) glycerol, 0.1 mg/ml BSA, 10% (w/v) polyethylene glycol 8000, 2.5 mM MgCl_2_, 200 nM DSB DNA substrate, consisting either of the upstream and downstream oligonucleotide duplexes (DSB.A, DSB.C, or DSB.D substrates, Fig. 2a) or an upstream single-strand primer oligonucleotide and the downstream duplex (DSB.B substrate, Fig. 2a) and full-length Polλ at 600 nM. Individual components of the reaction mix were added incrementally. First, Polλ was added to the annealed downstream duplex. Following a 5 min incubation at 37 °C, to allow the enzyme to bind the 5´-phosphorylated terminus of the downstream duplex, the annealed upstream portion of the DNA substrate was added, and the mixture was incubated for another 5 min. After this incubation, the reaction was initiated by the addition of dTTP to a final concentration of 5 µM. Ten microliter aliquots of the mixture were removed at different time points (as specified in Fig. 2b) and quenched with an equal volume of 99% formamide (v/v), 10 mM EDTA, and 0.1% (w/v) bromophenol blue. The products were resolved on a 16% (v/v) denaturing polyacrylamide gel, imaged using a Typhoon Imager (GE Healthcare), quantitated with ImageQuant TL, and graphed using GraphPad Prism (v. 9).

### Cell Lines

WT (C57BL/6) or *Polm*^*−/−*^ *Poll*^*−/−*^ double knock out murine fibroblast (MEF) cells (generously provided by Dr. L. Blanco) were derived from E14.5d embryos and immortalized by the introduction of SV40 large T-antigen as described^10^. MEF cells were maintained in DMEM supplemented with 10% (v/v) fetal bovine serum (FBS, Sigma), 100U/ml penicillin, 5 mM N-acetyl-l-cysteine (Sigma) at 37 ^°^C and 5% CO_2_. These lines were confirmed by qPCR to be free of mycoplasma contamination.

### Cell-based extrachromosomal NHEJ repair assay

Extrachromosomal DNA substrates were generated by annealing DNA strands (Ultramers, Integrated DNA Technologies) described in Supplementary Table 3 and electroporated with 600 ng of pMAX-GFP plasmid (Lonza) into 200,000 cells in a 10 μL volume with one 1350 V 30 ms pulse (Neon, Invitrogen) as described previously^10^, except that in the current study, two substrates were introduced in each electroporation. One substrate had partially complementary (Fig. 3e) or non-complementary end structures (Fig. 3g), thus engaging polymerase activity during NHEJ. A second ‘spike-in’ control substrate containing fully complementary 5ʹGC overhang ends (repaired by NHEJ independently of polymerase activity) was introduced in all experiments at 1/20^th^ the concentration of the polymerase-engaging substrates described above and was used to confirm consistent electroporation. Polymerase-deficient cells were complemented by the addition of 1 μg of the noted purified polymerases to the substrate transfection solution just before electroporation. Following transfection, cells were incubated with Benzonase mix (HBSS, 0.5 M MgCl_2_, Benzonase) at 37 ^°^C for 30 min. Cellular repair products were harvested using a QIAamp DNA mini kit (Qiagen). Each electroporation was reproduced in triplicate from three independent cell electroporations. Head-to-tail NHEJ repair products were amplified using 26 cycles using the primers 5′-TAAGCGATGCTCTCACCG-3′ and 5′- GATGGGTGTGAGAGTGAAGATC −3′, digested with restriction endonucleases specific for products of polymerase-dependent repair, as noted in Fig. 3f, h, and the fraction of polymerase-dependent repair determined by electrophoresis on a 10% (w/v) non-denaturing polyacrylamide gel, visualized using a Typhoon Imager (GE Healthcare). The intensities of digested and undigested bands were quantified using ImageQuant (v. 8.1), and resulting data were graphed and statistically analyzed using GraphPad Prism (v. 9).

### Crystallization of the 1nt-gapped SSB ternary complex

Synthesized DNA oligonucleotides (Integrated DNA Technologies) were annealed to generate a 1nt-gapped SSB substrate (Fig. 4b): unbroken template (5′-CGGCAGTACTG-3′), upstream primer (5′-CAGTAC)−3′ and 5′-phosphorylated downstream primer (5′-pGCCG-3′). Oligonucleotides were mixed in an equimolar ratio in 100 mM Tris pH 7.5, 40 mM MgCl_2_ and annealed in a thermal cycler, with denaturation at 94 °C, followed by a slow temperature gradient from 90 °C to 4 °C. The annealed DNA was then mixed in a 3:1 molar ratio with concentrated Polλ catalytic domain (Val235-Trp575, 14.47 mg/mL). The complex was incubated on ice at 4 °C for 1 hr, followed by the addition of the nonhydrolyzable incoming 2′-deoxyuridine-5′-[(α,β)-imido]triphosphate (dUMPNPP) nucleotide (0.91 mM final concentration). After another 1 h incubation at 4 °C, ternary complex crystals were grown at room temperature using the sitting drop vapor diffusion technique^26^. 300 nL of the protein/DNA complex were mixed with 300 nL of mother liquor (37.5 mM Na cacodylate pH 6.5, 150 mM KCl, 75 mM magnesium acetate, 7.5% (w/v) PEG 8000). The crystals were transferred from the drop to a solution containing 50 mM Na cacodylate pH 6.5, 200 mM KCl, 100 mM magnesium acetate, 50 mM NaCl, 10% (w/v) PEG 8000, and 1 mM dUMPNPP, then to a cryoprotectant (50 mM Na cacodylate pH 6.5, 200 mM KCl, 100 mM magnesium acetate, 50 mM NaCl, 20% (w/v) PEG 8000, 15% (v/v) ethylene glycol) in two steps. Crystals were flash frozen in liquid nitrogen.

### Crystallization of complementary DSB.A quaternary complex

DNA oligonucleotides were used to generate the complementary DSB substrate (Fig. 5a): upstream template (5′-ACTG-3′), upstream primer (5′-CAGTGC)−3′, downstream template (5′-CGGCAGC-3′) and 5′-phosphorylated downstream primer (5′-pGCCG-3′), where the template strand break lies between the −2 and the −3 positions. Upstream and downstream DNA mixtures were separately annealed in a thermal cycler, as for the 1nt-gapped SSB substrate. The annealed DNA was then serially mixed in a 2.3:1 molar ratio with concentrated Polλ catalytic domain (Val235-Trp575, 13.7 mg/mL)—first the downstream DNA, followed by the upstream DNA, and finally the incoming nonhydrolyzable dUMPNPP nucleotides (0.91 mM final concentration). The complex was incubated on ice at room temperature for 1 h, after each addition. Crystals of the quaternary complex were grown at 4 °C using the sitting drop vapor diffusion technique^26^. 400 nL of the protein/DNA complex were mixed with 200 nL of mother liquor (0.192 M tri-potassium citrate, 19.2% (w/v) PEG 3350). The growth drops were opened, and 500 nL of 0.188 M tri-potassium citrate, 18.8% (w/v) PEG 3350, 10 mM MgCl_2_, 0.5 mM dUMPNPP were added, followed by four subsequent 500 nL additions of cryoprotectant solution containing 20 mM Tris pH 8, 50 mM NaCl, 0.2 M tri-potassium citrate, 20% (w/v) PEG 3350, 25% (v/v) ethylene glycol, 10 mM MgCl_2_, 0.5 mM dUMPNPP, with 30-60 sec incubation between additions. Crystals were looped directly from this drop and flash frozen in liquid nitrogen.

### Crystallization of pre-catalytic DSB.B synaptic complex

DNA oligonucleotides were used to generate the DSB.B substrate (Fig. 6a): upstream primer (5′-CAGTGC)-3′, downstream template (5′-CGGCAGC-3′) and 5′-phosphorylated downstream primer (5′-pGCCG-3′). Upstream and downstream DNA mixtures were separately annealed in a thermal cycler, as for the 1nt-gapped SSB substrate. The annealed DNA was then serially mixed in a 2.3:1 molar ratio with concentrated Polλ catalytic domain (Pro233-Trp575, 13.7 mg/mL)—first the downstream DNA duplex, followed by the upstream primer, and finally the incoming nonhydrolyzable dUMPNPP nucleotide (0.91 mM final concentration). The complex was incubated on ice at 4 °C for 1 h, after each addition. Crystals of the quaternary complex were grown were grown at room temperature using the sitting drop vapor diffusion technique^26^. 300 nL of the protein/DNA complex were mixed with 300 nL of mother liquor (0.2 M sodium sulfate, 20% (w/v) PEG 3350). The growth drops were opened and 500 nL of mother liquor was added, followed by three subsequent 500 nL additions of cryoprotectant solution containing 0.138 M sodium sulfate, 13.8% (w/v) PEG 3350, 20 mM MgCl_2_, 25% (v/v) ethylene glycol, 0.5 mM dUMPNPP. The crystals were then transferred from the drop directly to the cryoprotectant solution, then flash frozen in liquid nitrogen.

### Crystallization of mismatched DSB.C quaternary complex

DNA oligonucleotides were used to generate the DSB substrate containing a mispair upstream of the primer terminus (Fig. 7a): upstream template (5′-**T**ACTG-3′), upstream primer (5′-CAGT**G**C)−3′, downstream template (5′-CGGCAG-3′) and 5′-phosphorylated downstream primer (5′-pGCCG-3′), where the template strand break lies between the −1 and −2 positions, and mispaired bases at the −2 position are indicated in bold. Upstream and downstream DNA mixtures were separately annealed in a thermal cycler, as for the 1nt-gapped SSB substrate. The annealed DNA was then serially mixed in a 2.3:1 molar ratio with concentrated Polλ catalytic domain (Val235-Trp575, 14.47 mg/mL)—first the downstream DNA, followed by the upstream DNA, and finally the incoming nonhydrolyzable dUMPNPP nucleotides (0.91 mM final concentration). The complex was incubated on ice at 4 °C for 1 hr, after each addition. Crystals of the quaternary complex were grown at 4 °C using the sitting drop vapor diffusion technique^26^. 400 nL of the protein/DNA complex were mixed with 200 nL of mother liquor (76.5 mM Na cacodylate pH 6.5, 0.153 M ammonium sulfate, 22.95% (w/v), PEG 8 K, 13.5% (v/v) glycerol, 0.2 M NDSB-201). The crystals were transferred from the drop to a cryoprotectant solution containing 76.5 mM Na cacodylate pH 6.5, 0.153 M ammonium sulfate, 22.95% (w/v), PEG 8 K, 13.5% (v/v) glycerol, 20 mM MgCl_2_, 80 mM NDSB-201, and 0.5 mM dUMPNPP. Crystals were flash frozen in liquid nitrogen.

### Crystallization of blunt-ended DSB.D quaternary complex

DNA oligonucleotides were used to generate the blunt-end DSB substrate (Fig. 8a): upstream template (5′-GCACTG-3′), upstream primer (5′-CAGTGC)−3′, downstream template (5′-CGGCA-3′) and 5′-phosphorylated downstream primer (5′-pGCCG-3′). Upstream and downstream DNA mixtures were separately annealed in a thermal cycler, as for the 1nt-gapped SSB substrate. The annealed DNA was then serially mixed in a 2.3:1 molar ratio with concentrated Polλ catalytic domain (Val235-Trp575, 14.47 mg/mL)—first the downstream DNA, followed by the upstream DNA, and finally the incoming nonhydrolyzable dUMPNPP nucleotide (0.91 mM final concentration). The complex was incubated on ice at 4 °C for 1 hr, after each addition. Crystals of the quaternary complex were grown at 4 °C using the sitting drop vapor diffusion technique^26^. 500 nL of the protein/DNA complex were mixed with 100 nL of mother liquor (80 mM Na cacodylate pH 6.5, 0.16 M calcium acetate, 14.4% (w/v) PEG 8 K, 20% (v/v) glycerol). The crystals were transferred from the drop to a cryoprotectant solution containing 76 mM Na cacodylate pH 6.5, 0.152 M magnesium acetate, 15.2% (w/v), PEG 8 K, 19% (v/v) glycerol, and 0.5 mM dUMPNPP and soaked for 27 h at 4 °C. After soaking for 3 h, crystals were transferred to cryprotectant solution containing 76 mM Na cacodylate pH 6.5, 0.152 M magnesium acetate, 15.2% (w/v), PEG 8 K, 19% (v/v) glycerol, and 5 mM dTTP and soaked for 24 h at 4 °C, yielding a mixture of incorporated and unincorporated dTTP within the crystal. Crystals were flash frozen in liquid nitrogen.

### Data collection and structural refinement

Crystals were placed into a stream of nitrogen gas cooled to −180 °C for data collection. Data were collected on the Southeast Regional Collaborative Access Team (SER-CAT) 22-ID beamline at the Advanced Photon Source (APS) at Argonne National Laboratory. The data were integrated, and scaled using HKL2000^27^ (v. 719.2). The crystal structure of the previously reported pre-catalytic 1nt-gapped SSB ternary complex (PDB ID code 2PFO^18^) was used as the search model for the molecular replacement of the current SSB complexes in Phaser^28^ (v. 2.8.3). The structure of the current pre-catalytic ternary SSB complex (PDB ID code 7M07) was used as the molecular replacement model for all other complexes. In order to decrease potential model bias, the same *R*_*free*_ test set was used for all structures with this space group and unit cell dimensions. All structures were refined by iterative cycles of manual model building and refinement in COOT^29,30^ (v. 0.8.9.2) and Phenix^31^ (v. 1.19_4092). TLS (Translation/Libration/Screw) vibrational motion refinement^32^ was used for all structures. Data collection and refinement statistics are listed in Supplementary Table 1. Ramachandran statistics were generated using MolProbity^33^. All superpositions and structural figures were generated using PyMOL (v. 2.2.0).

### Reporting summary

Further information on research design is available in the Nature Research Reporting Summary linked to this article.

## Supplementary information

Supplementary Information

Reporting Summary

## Data Availability

The data that support this study are available from the corresponding author upon reasonable request. Atomic coordinates and structure factors have been deposited in the Protein Data Bank (https://www.rcsb.org/) with ID codes 7M07, 7M09, 7M0A, 7M0B, 7M0D, 7M0E. Source data are provided with this paper.

## Source data

Source Data
