# Supplementary material for: Analysis of diverse double-strand break synapsis with Polλ reveals basis for unique substrate specificity in nonhomologous end-joining
Source: Nat Commun. 2022 Jul 1;13:3806. doi: 10.1038/s41467-022-31278-4 (PMC9249759; doi:10.1038/s41467-022-31278-4)
Supplement: Supplementary file 1 — Supplementary Information [file 41467_2022_31278_MOESM1_ESM.pdf]

Analysis of diverse double-strand break synapses with Polλ reveals basis for unique substrate specificity in nonhomologous end-joining

Andrea M. Kaminski[1], Kishore K. Chiruvella[2], Dale A. Ramsden[2], Katarzyna Bebenek[1*], Thomas A. Kunkel[1], Lars C. Pedersen[1]

[1]Genome Integrity and Structural Biology Laboratory, National Institute of Environmental Health Sciences, National Institutes of Health, 111 TW Alexander Dr., Bldg. 101, Research Triangle Park, North Carolina, USA, 27709.
[2]Department of Biochemistry and Biophysics, Lineberger Comprehensive Cancer Center, University of North Carolina at Chapel Hill, Chapel Hill, NC 27599.

Correspondence: bebenek@niehs.nih.gov

PDF Contents:

Supplementary Fig. 1. Electron density maps for Polλ/DNA complexes presented in this study

Supplementary Fig. 2. Structural characterization of the Polλ 'brooch' motif

Supplementary Fig. 3. Composition of the asymmetric unit for the DSB.A fully complementary complex

Supplementary Fig. 4. Composition of the asymmetric unit for the DSB.B mispaired synaptic complex

Supplementary Fig. 5. Expanded view of individual mispairs of the DSB.B synaptic complex

Supplementary Table 1. Data collection and refinement statistics

Supplementary Table 2. Map of structurally-equivalent interactions for the Polλ SSB and DSB Complexes

Supplementary Table 3. Oligonucleotide substrates used in the cell-based NHEJ assay

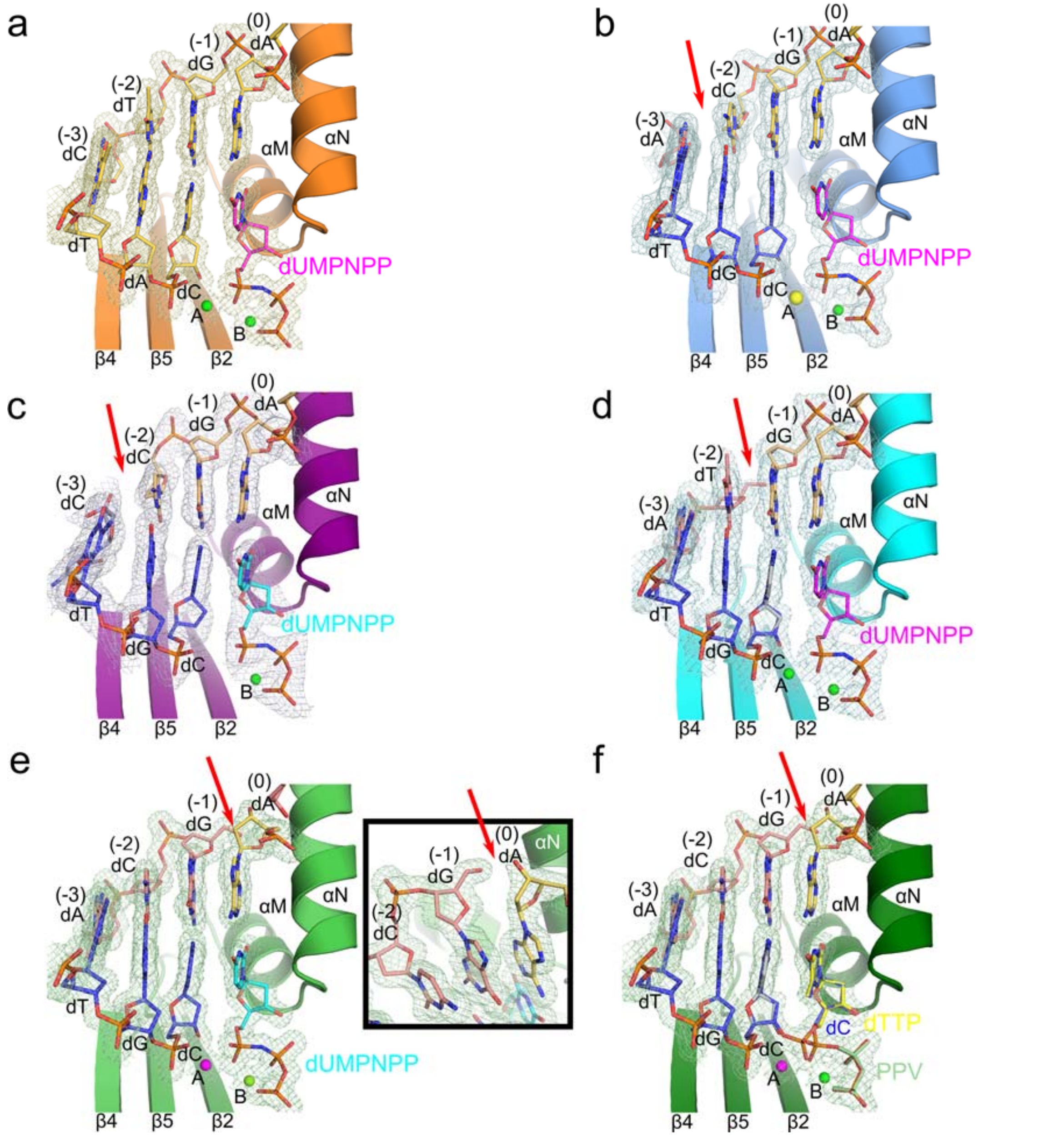

Supplementary Figure 1: Electron density maps for Polλ/DNA complexes presented in this study. Structures of the SSB and DSB substrates bound in the active site of the Polλ catalytic domain. **a**. Pre-catalytic ternary complex with SSB substrate. **b**. Pre-catalytic quaternary complex with DSB.A (break site between -3 and -2 positions). **c**. Pre-catalytic DSB.B synaptic complex (break site between -3 and -2 positions), with largely mispaired upstream duplex. **d**. Pre-catalytic quaternary DSB.C complex with break site between the -2 and -1 positions, and G:T mispair proximal to the break site. **e**. Pre-catalytic quaternary complex with blunt-ended DSB.D, with zoomed-in view of the template strand break site (inset). **f**. Partially incorporated complex with DSB.D. Key protein secondary structural elements shown in cartoon for all structures, and the DNA substrates and incoming nucleotides (dTTP in yellow, dUMPNPP in cyan or magenta, as indicated) are drawn in stick. Ions bound in the active site are represented by spheres (Mg^{2+} in green, Ca^{2+} in magenta, and Na^{+} in yellow). The $2F_o$-F_c electron density map (mesh, contoured at 1σ) is shown with the SSB or DSB bound in the active site of each complex.

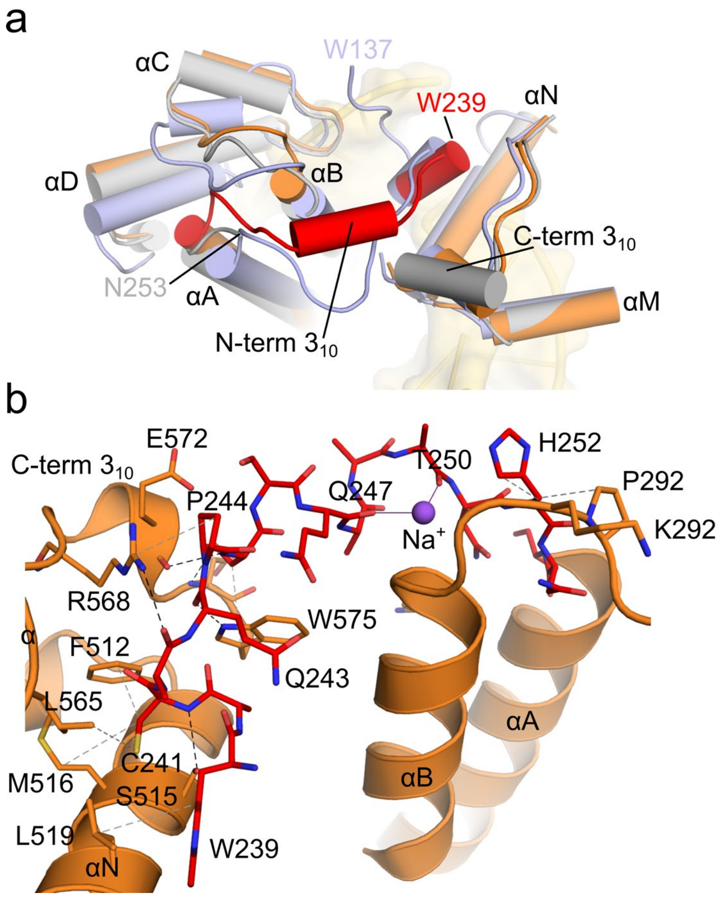

Supplementary Figure 2: Structural characterization of the Polλ 'brooch' motif. **a**. Superposition of the 'brooch' motifs (residues Val235-Asn252) of Polλ, (orange, 'brooch' in red) and Polμ (PDB ID code 4M04[14], light blue) with the same region in Polλ without the 'brooch' (PDB ID code 2PFO[13], gray). The position of the N-terminal ordered residue in each structure has been marked in each structure's corresponding color. The 'brooch' in Polλ is ordered beginning with Trp239 and lies within a cleft between the 8 kDa and thumb subdomains Small differences are observed in positioning the N-terminal end of α-helix A, and in the loop between α-helices B and C. The 'brooch' motif in Polλ occupies the same cleft as in human Polμ and mouse TdT (PDB ID code 1JMS[15]), but adopts a different conformation. Superposition of the Polλ ternary complexes with that of Pol μ shows that both structures contain a short α-helix, which lies near α-helix N, but Polλ also contains a short, slightly distorted 3_{10} helix proximal to the C-terminal 3_{10} helix. **b**. Analysis of interactions involving the 'brooch' motif (red) indicates that this structural feature covers a hydrophobic patch on the Polλ catalytic domain (orange) comprised of nonpolar residues from α-helices O and N and the C-terminal 3_{10} helix. Therefore, the interactions of the 'brooch' with this region are largely driven by Van der Waals forces (indicated by gray dashed lines). There are a few putative hydrogen bonding interactions (black dashed lines) which are primarily mediated by backbone atoms (Trp239 O – Cys241 N, Trp575 NE1 – Gln243 O, Ser245 N – Glu572 O), and only a single hydrogen bond involving a 'brooch' motif sidechain (Ser245 OG – Asp574 N). Coordination of the Na^+ ion (purple sphere) is shown in solid purple lines.

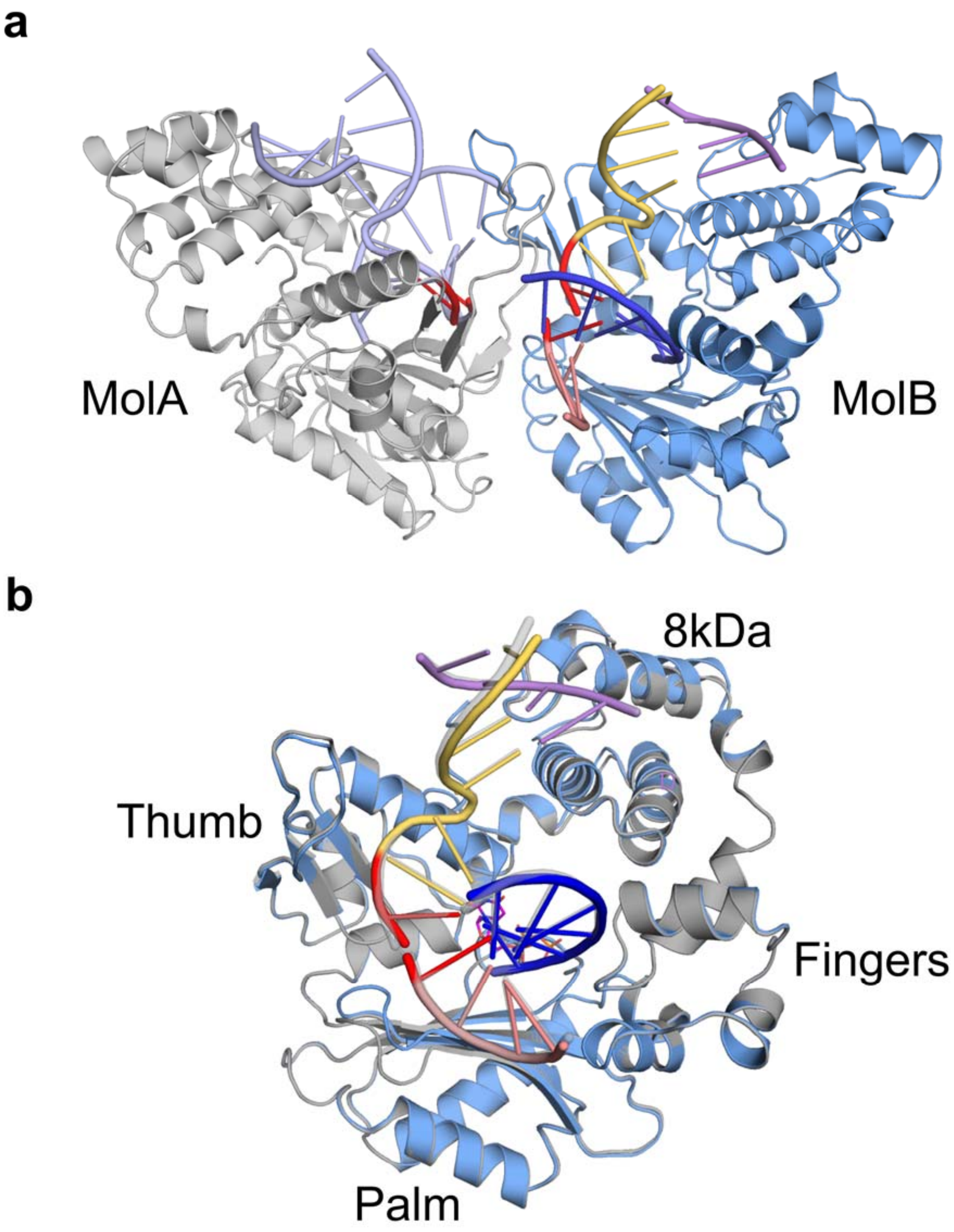

Supplementary Figure 3: Composition of the asymmetric unit for the DSB.A fully complementary complex. a. Ribbon diagram of the two molecules of the Polλ pre-catalytic DSB.A quaternary complex within the asymmetric unit. The catalytic domain of Polλ (molecule A in gray; molecule B in light blue) with their bound DNA substrates (bound to molecule A in blue gray; bound to molecule B, upstream template--chain I—and primer—chain J—in pink and blue, respectively, and downstream template—chain K—and primer—chain L—in light orange and lavender, respectively). **b**. Structural superposition of the two DSB.A quaternary complexes in the asymmetric unit (molecule A in gray, DNA in transparent gray; molecule B in light blue, DNA colored as in **a**).

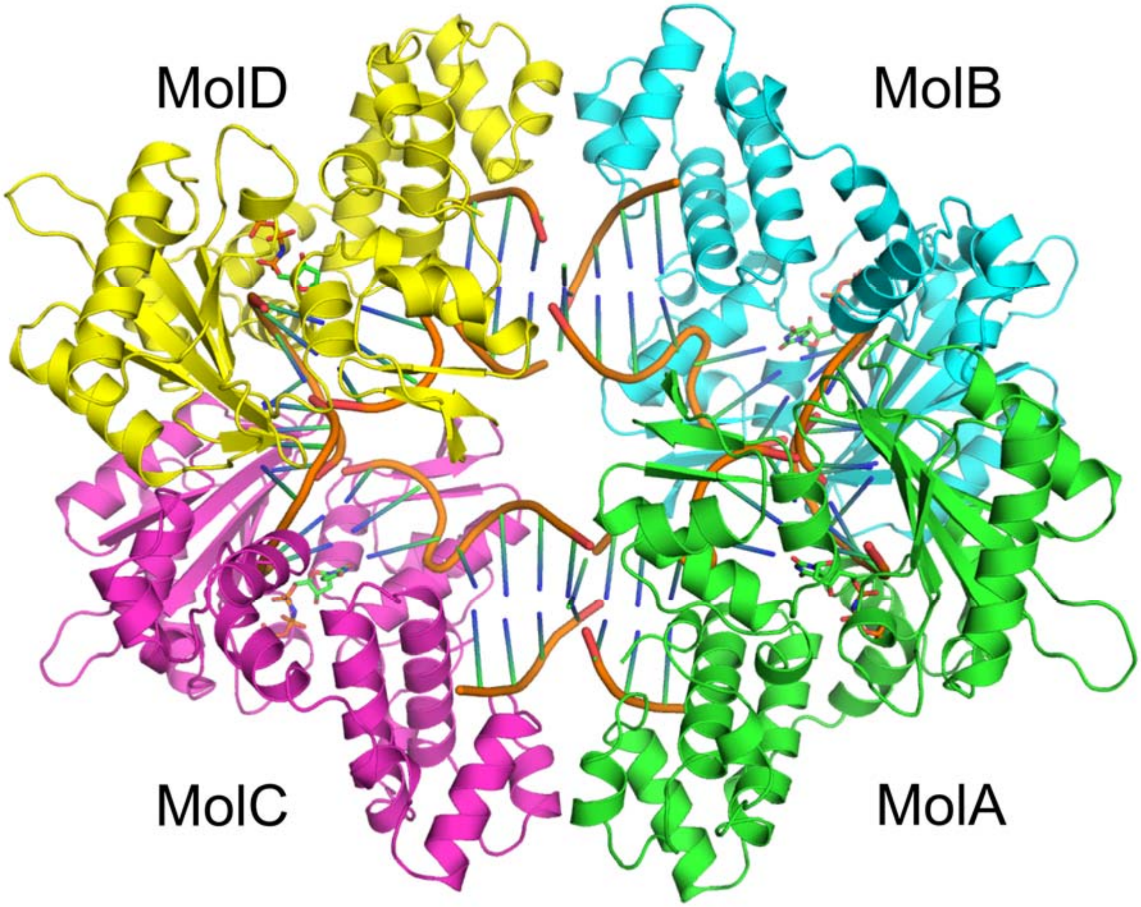

Supplementary Figure 4: Composition of the asymmetric unit for the DSB.B mispaired synaptic complex. Ribbon diagram of the asymmetric unit for the Polλ pre-catalytic mispaired DSB.B synaptic complex. The upstream primer for molecule A (green) extends towards toward the active site of molecule B (cyan), and vice versa. This configuration also holds true for the upstream primers of molecules C (magenta) and D (green). Dynamic pseudo-stacking between the DNA ends bound to molecules D and B as well as molecules A and C is modeled with alternate conformations.

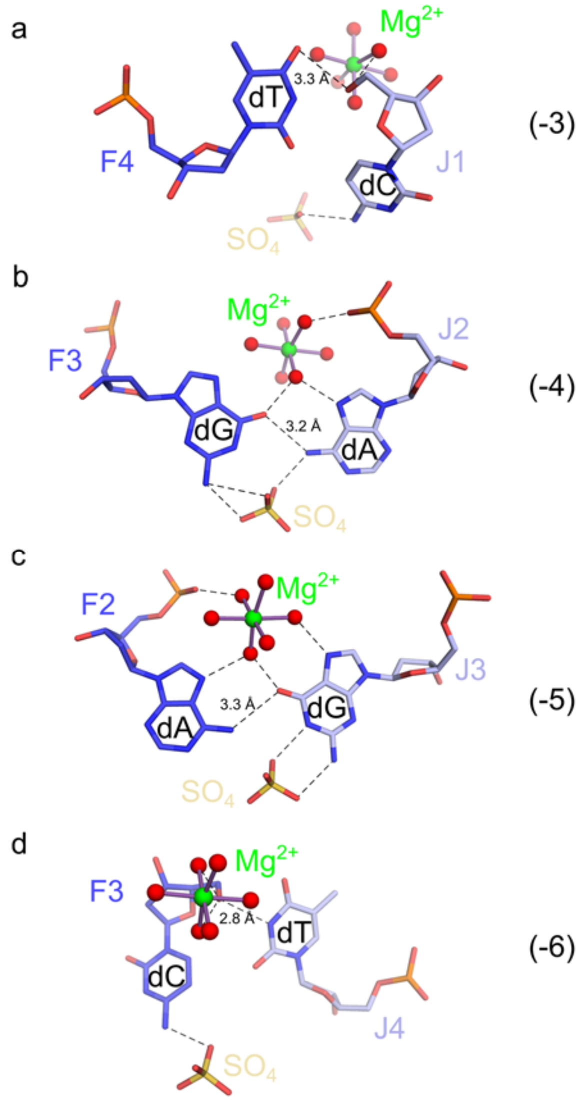

Supplementary Figure 5: Expanded view of individual mispairs of the DSB.B synaptic complex. Base mispairs within the upstream duplex, from the -3 position (**a**), closest to the active site, stepping upstream through the -4 (**b**), -5 (**c**), and -6 (**d**) positions. Each pairing is drawn in stick, with the upstream primer in blue, and the surrogate upstream template from the neighboring molecule in light blue. The sulfate (yellow) and Mg^{2+} ions (green, with coordinating water molecules, red spheres) are also shown. Putative hydrogen bonding interactions are indicated by black dashed lines and interatomic distances of bonds between the bases are given. The base mispair (T:C) at the -3 position (**a**), immediately upstream of the break site deviates substantially from cognate Watson-Crick geometry and is instead maintained only by a putative hydrogen bond between the 5′-OH on template residue J1 and O4 of primer residue F4. The 5′-OH also interacts with one of the water molecules in the hydration sphere of the Mg^{2+}ion. A putative hydrogen bond is observed between the N4 atom on J1 and a sulfate ion. The bases of the next mispair upstream (G:A at the -4 position, **b**) are slightly offset from one another, with interactions largely mediated by the Hoogsteen surface of each. Only a single hydrogen bond is observed between the bases (F3 O6 – J2 N6), but a network of other interactions are observed, involving the hydrated Mg^{2+} and sulfate ions. The same trend holds true for the reciprocal A:G mispair at the -5 position (F2 N6 – J3 O6, **d**). The C:T mispair (-6 position, **d**) is the reciprocal of the T:C mispair at the -3 position and makes no direct hydrogen bonds between the bases. The positioning of this pair involves only the Mg^{2+} and sulfate ions.

Supplementary Table 1: Data collection and refinement statistics

	SSB pre-catalytic ternary complex[a,b]	DSB.A pre-catalytic quaternary complex[a,b]	DSB.B pre-catalytic synaptic complex[a,b]
PDB ID code	7M07	7M0D	7M0E
**Data collection**			
Space group	$P2_12_12_1$	$P2_12_12$	P1
Cell dimensions			
a, *b*, *c* (Å)	56.00, 59.67, 139.65	95.44, 151.99, 86.47	64.97, 85.82, 91.42
A, β, γ (°)	90, 90, 90	90, 90, 90	105.22, 92.58, 11.30
Resolution (Å)	50-1.57 (1.60-1.57)[c]	50-1.80 (1.83-1.80)	50-2.25 (2.29-2.25)
R_{sym} (%)	9.6 (50)	10.2 (92.7)	9.2 (84.8)
Mean $I / \sigma I$	26.8 (1.75)	17.70 (2.07)	17.4 (1.73)
Completeness (%)	99.9 (99.1)	99.1 (100)	91.9 (93.6)
Redundancy	6.5 (4.5)	6.5 (7.3)	3.8 (3.7)
**Refinement**			
Resolution (Å)	45.36-1.57	41.78-1.80	38.16-2.25
No. reflections	65909	115456	75728
R_{work} / R_{free} (%)	17.05/18.77	16.43/18.87	18.54/22.21
No. atoms			
Protein	2553	2664/2691	2534/2446/2449/2415
DNA	464	423/423	344/344/344/344
Nucleotide	28[e]	28/28[e]	28/28/28/28[e]
Water	445	1091	354
B-factors			
Protein	26.57	20.43/20.32	38.40/45.99/49.90/64.35
DNA	22.04	25.80/24.78	37.62/43.61/44.88/54.37
Nucleotide	14.06[e]	12.74/12.05	24.97/26.66/33.60/44.45[e]
Water	33.06	32.34	42.06
R.m.s. deviations			
Bond lengths (Å)	0.010	0.009	0.005
Bond angles (°)	0.986	1.033	0.681
Ramachandran favored (%)	99.07	98.96	97.94

[a]A single crystal was used to collect each data set
[b]Data were collected on the Southeast Regional Collaborative Access Team (SER-CAT) 22-ID beamline at the Advanced Photon Source at Argonne National Laboratory.
[c]Values in parentheses are for highest-resolution shell.
[d]Includes atoms from unincorporated and unincorporated alternate conformations of residues P6 and P7.
[e]Nonhydrolyzable incoming dUMPNPP nucleotide.

Supplementary Table 1: Data collection and refinement statistics (continued)

	DSB.C pre-catalytic quaternary complex[a,b]	DSB.D pre-catalytic quaternary complex[a,b]	DSB.D incomplete incorporation complex[a,b]
PDB ID code	7M0B	7M09	7M0A
**Data collection**			
Space group	$P2_12_12_1$	$P2_12_12_1$	$P2_12_12_1$
Cell dimensions			
a, *b*, *c* (Å)	56.37, 59.96, 139.41	55.94, 59.62, 140.04	55.77, 59.57, 140.73
Α, β, γ (°)	90, 90, 90	90, 90, 90	90, 90, 90
Resolution (Å)	50-2.0 (2.03-2.00)[c]	50-1.65 (1.68-1.65)	50-1.83 (1.86-1.83)
R_{sym} (%)	15.3 (81.9)	6.7 (80.3)	7.2 (70.3)
Mean I / σI	13.3 (1.55)	16.94 (1.31)	18.27 (1.62)
Completeness (%)	99.2 (98.4)	99.8 (99.9)	99.0 (98.9)
Redundancy	5.9 (4.3)	6.5 (6.1)	6.7 (6.6)
**Refinement**			
Resolution (Å)	39.40-2.00	39.17-1.65	43.71-1.83
No. reflections	32401	57346	41719
R_{work} / R_{free} (%)	17.05/20.04	16.77/18.61	16.90/19.33
No. atoms			
Protein	2580	2618	2624
DNA	443	423	462[d]
Nucleotide	28[e]	28[e]	28/9[d]
Water	385	457	409
B-factors			
Protein	24.41	24.66	25.61
DNA	21.89	22.08	24.14
Nucleotide	12.48[e]	13.57[e]	19.51[e]/23.75[f]
Water	32.12	34.13	35.20
R.m.s. deviations			
Bond lengths (Å)	0.080	0.010	0.006
Bond angles (°)	0.938	1.035	0.772
Ramachandran favored (%)	98.47	98.78	98.16

[a]A single crystal was used to collect each data set
[b]Data were collected on the Southeast Regional Collaborative Access Team (SER-CAT) 22-ID beamline at the Advanced Photon Source at Argonne National Laboratory.
[c]Values in parentheses are for highest-resolution shell.
[d]Includes atoms from unincorporated and unincorporated alternate conformations of residues P6 and P7.
[e]Nonhydrolyzable incoming dUMPNPP nucleotide.
[f]Inorganic pyrophosphate leaving group.

Supplementary Table 2: Map of structurally-equivalent interactions for the Polλ SSB and DSB Complexes

SSB		DSB.A		DSB.B		DSB.C		DSB.D	
**Interaction**	**Dist. (Å)**	**Interaction**	**Dist. (Å)**	**Interaction**	**Dist. (Å)**	**Interaction**	**Dist. (Å)**	**Interaction**	**Dist. (Å)**
Lys521 NZ - T4 OP1	2.6			Lys521 NZ - G4 OP1	2.8	Lys521 NZ - T4 OP1	2.9	Lys521 NZ - T4 OP1	2.7
								Lys521 NZ - T3 O3′	3.1
		Lys521 NZ - K6 OP2	2.8						
Arg514 NE - T5 OP2	2.9	Arg514 NE - K5 OP2	2.9	Arg514 NE - G5 OP2	2.9	Arg514 NE - T5 OP2	2.9	Arg514 NE - T5 OP2	3
Arg514 NH2 - OP1	3.2	Arg514 NH2 - K5 OP1	3	Arg514 NH2 - G5 OP1	3	Arg514 NH2 – T5 OP1	3	Arg514 NH2 - T5 OP1	2.8
Arg517 NH1 - T5 N3	3.3					Arg517 NH1 - T5 N3	3.1	Arg517 NH1 - T5 N3	3.1
Arg517 NH1 - T6 N3	3	Arg517 NH1 - K6 N3	3.1	Arg517 NH1 - G6 N3	3.1	Arg517 NH1 - T6 N3	3	Arg517 NH1 - U1 N3	3.1
Agr517 NH1 - T6 O4′	3.16	Arg517 NH1 - K6 O4′	3.3	Arg517 NH1 - G6 O4′	2.9	Arg517 NH1 - T6 O4′	3.1	Arg517 NH1 - U1 O4′	3.3
								Leu527 O - U1 O5′	2.8
Glu529 OE1 - Arg517 NH2	3.1	Glu529 OE1 - Arg517 NH2	2.8	Glu529 OE2 - Arg517 NH2	2.9				
				Glu529 OE2 - Arg517 NH1	3.1				
Glu529 OE2 - T6 N2	3.1	Glu529 OE2 - K6 N2	3.2	Glu529 OE2 - G6 N2	3				
		Glu529 OE2 - K7 O3′	2.8	Glu529 OE1 - G7 O3′	2.6				
		Lys544 NZ - K7 OP2	3.3						
His530 NE2 - T8 OP1	2.8					His530 NE2 - U2 OP1	2.8	His530 NE2 - U3 OP1	2.8
		His530 NE2 - K7 O3′	3	His530 NE2 - G7 O3′	2.9				
		His530 NE2 - Asn467 OD1	2.9	His530 NE2 - Asn467 OD1	2.9				
		Asn467 OD1 - K7 O3′	3						
		Asn467 ND2 - Gln464 OE1	3.1						
Gln471 NE2 - T8 OP1	3	Glu465 N - I2 OP1	3.2			Gln471 NE2 - U2 OP1	3.2	Gln471 NE2 - T8 OP1	3
Gln471 N - T9 OP1	2.9	Glu466 N - I2 OP1	2.9			Gln471 N - U3 OP1	3	Gln471 N - U4 OP1	2.8
Lys472 N - T9 OP1	3.2					Lys472 N - U3 OP1	3.3	Lys472 N - U4 OP1	3.1
						Lys472 NZ - U2 N3	3.3	Lys472 NZ - U3 O3′	3
				Lys472 NZ - J1 O2	2.8				
				Glu465 OE2 - J1 N3	2.5				
				Glu465 OE1 - J1 N4	3.1				
		Ser463 N - I3 OP1	3						
		Gln470 NE2 - Glu529 O	3	Gln470 NE2 - Glu529 O	3.2				
		Gln471 NE2 - Glu465 OE1	3						
				Gly468 N - J3 OP1	2.8				

Supplementary Table 3: Oligonucleotide substrates used in the cell-based NHEJ assay

**Oligo Position**	**Sequence**
GCG3′ Top strand (Fig. 3e-f)	5′pTTAGCTGTATAGTCACCCTGCAGATCTTCACTCTCACACCCATCGCACGATTCACTCTGGCAGTGCTATTGGGACTTCGGCTGAGGAGGACACACTGCACTTGTGGTGGATGACCTAAGCGATGCTCTCACCGAGAGAAGCAGGGTAGCCAGTCTGAGAAGCG3′
GCG3′ Bottom strand (Fig. 3e-f)	5′pTTCTCAGACTGGCTACCCTGCTTCTCTCGGTGAGAGCATCGCTTAGGTCATCCACCACAAGTGCAGTGTGTCCTCCTCAGCCGAAGTCCCAATAGCACTGCCAGAGTGAATCGTGCGATGGGTGTGAGAGTGAAGATCTGCAGGGTGACTATACAGCTAAGCG3′
Blunt/CG, Top strand (Fig. 3g-h)	5′pTGTTAGCTGTATAGTCACCCTGCAGATCTTCACTCTCACACCCATCGCACGATTCACTCTGGCAGTGCTATTGGGACTTCGGCTGAGGAGGACACACTGCACTTGTGGTGGATGACCTAAGCGATGCTCTCACCGAGAGAAGCAGGGTAGCCAGTCTGAGACAGC3′
Blunt/CG, Bottom strand (Fig. 3g-h)	5′pTGTCTCAGACTGGCTACCCTGCTTCTCTCGGTGAGAGCATCGCTTAGGTCATCCACCACAAGTGCAGTGTGTCCTCCTCAGCCGAAGTCCCAATAGCACTGCCAGAGTGAATCGTGCGATGGGTGTGAGAGTGAAGATCTGCAGGGTGACTATACAGCTAACA3′
5′GC Top strand (Fig. 3f and h)	5′pGCTGAAGGACTGTTGCGTGCACGATTCACTCTGTTCCATGTCCAAGATACGGATCTTCACTCTCACACCCATCGATGGGACTTCGGCTGAGGAGGACATGTTAGACTTGTGGTGGATGACTAAGCGATGCTCTCACCGAAGTGTCAGTCTTCATCAAGGTCACGCGTGACT3′
5′GC Bottom strand (Fig. 3f and h)	5′pGCAGTCACGCGTGACCTTGATGAAGACTGACACTTCGGTGAGAGCATCGCTTAGTCATCCACCACAAGTCTAACATGTCCTCCTCAGCCGAAGTCCCATCGATGGGTGTGAGAGTGAAGATCCGTATCTTGGACATGGAACAGAGTGAATCGTGCACGCAACAGTCCTTCA3′
