# Supplementary material for: Analysis of diverse double-strand break synapsis with Polλ reveals basis for unique substrate specificity in nonhomologous end-joining
Source: Nat Commun. 2022 Jul 1;13:3806. doi: 10.1038/s41467-022-31278-4 (PMC9249759; doi:10.1038/s41467-022-31278-4)
Supplement: Supplementary file 2 — Reporting Summary [file 41467_2022_31278_MOESM2_ESM.pdf]

nature research

Corresponding author(s): Katarzyna Bebenek

Last updated by author(s): Mar 14, 2022

Reporting Summary

Nature Research wishes to improve the reproducibility of the work that we publish. This form provides structure for consistency and transparency in reporting. For further information on Nature Research policies, see our Editorial Policies and the Editorial Policy Checklist.

Statistics

For all statistical analyses, confirm that the following items are present in the figure legend, table legend, main text, or Methods section.

n/a	Confirmed	
☐	☒	The exact sample size (*n*) for each experimental group/condition, given as a discrete number and unit of measurement
☐	☒	A statement on whether measurements were taken from distinct samples or whether the same sample was measured repeatedly
☒	☐	The statistical test(s) used AND whether they are one- or two-sided<br>*Only common tests should be described solely by name; describe more complex techniques in the Methods section.*
☒	☐	A description of all covariates tested
☒	☐	A description of any assumptions or corrections, such as tests of normality and adjustment for multiple comparisons
☐	☒	A full description of the statistical parameters including central tendency (e.g. means) or other basic estimates (e.g. regression coefficient) AND variation (e.g. standard deviation) or associated estimates of uncertainty (e.g. confidence intervals)
☒	☐	For null hypothesis testing, the test statistic (e.g. *F*, *t*, *r*) with confidence intervals, effect sizes, degrees of freedom and *P* value noted<br>*Give P values as exact values whenever suitable.*
☒	☐	For Bayesian analysis, information on the choice of priors and Markov chain Monte Carlo settings
☒	☐	For hierarchical and complex designs, identification of the appropriate level for tests and full reporting of outcomes
☒	☐	Estimates of effect sizes (e.g. Cohen's *d*, Pearson's *r*), indicating how they were calculated

Our web collection on statistics for biologists contains articles on many of the points above.

Software and code

Policy information about availability of computer code

Data collection	The data were integrated, and scaled using HKL2000 (v. 719.2). The phase problem was solved by molecular replacement in Phaser (v. 2.8.3). All structures were refined by iterative cycles of manual model building and refinement in COOT (v. 0.8.9.2) and Phenix (v. 1.19_4092). TLS (Translation/Libration/Screw) vibrational motion refinement was used for all structures.
Data analysis	Ramachandran statistics for each structure were generated by MolProbity. All superpositions and structural figures were generated using PyMOL (v. 2.2.0). Quantitation of the in vitro DSB activity assays was performed using ImageQuant TL and graphed using GraphPad Prism (v.9.3.0). Quantitation of the cell-based NHEJ assays was performed using ImageQuant (v. 8.1) and graphed using GraphPad Prism (v. 9).

For manuscripts utilizing custom algorithms or software that are central to the research but not yet described in published literature, software must be made available to editors and reviewers. We strongly encourage code deposition in a community repository (e.g. GitHub). See the Nature Research guidelines for submitting code & software for further information.

Data

Policy information about availability of data

All manuscripts must include a data availability statement. This statement should provide the following information, where applicable:

- Accession codes, unique identifiers, or web links for publicly available datasets
- A list of figures that have associated raw data
- A description of any restrictions on data availability

Atomic coordinates and structure factors have been deposited in the Protein Data Bank (www.pdb.org) with ID codes 7M07, 7M09, 7M0A, 7M0B, 7M0D, 7M0E. Source data are provided with this paper, for Figures 2b, 2c , 3f, and 3h, and are included in the Source Data file.

Field-specific reporting

Please select the one below that is the best fit for your research. If you are not sure, read the appropriate sections before making your selection.

- [x] Life sciences
- [ ] Behavioural & social sciences
- [ ] Ecological, evolutionary & environmental sciences

For a reference copy of the document with all sections, see nature.com/documents/nr-reporting-summary-flat.pdf

Life sciences study design

All studies must disclose on these points even when the disclosure is negative.

Sample size	In vitro DSB activity reactions were prepared as biological replicates, with n=6 for DSB substrates A-C, and n=4 for DSB substrate D. Each of the cell-based NHEJ assays were performed as independent electroporations, in triplicate.
Data exclusions	No data were excluded from either the in vitro DSB activity assays or the cell-based NHEJ assays.
Replication	In vitro DSB activity reactions were prepared as biological replicates, with n=6 for DSB substrates A-C, and n=4 for DSB substrate D. Each of the cell-based NHEJ assays were performed as independent electroporations, in triplicate.
Randomization	Randomization was not appropriate for this study, since these are not population-based or clinical studies. The assays in this study were to determine biochemical activity only, and were quantitative, rather than subjective.
Blinding	Blinding was not appropriate for this study, since these are not population-based or clinical studies. The assays in this study were to determine biochemical activity only, and were quantitative, rather than subjective.

Reporting for specific materials, systems and methods

We require information from authors about some types of materials, experimental systems and methods used in many studies. Here, indicate whether each material, system or method listed is relevant to your study. If you are not sure if a list item applies to your research, read the appropriate section before selecting a response.

Materials & experimental systems

n/a	Involved in the study	
x		Antibodies
	x	Eukaryotic cell lines
x		Palaeontology and archaeology
x		Animals and other organisms
x		Human research participants
x		Clinical data
x		Dual use research of concern

Methods

n/a	Involved in the study	
x		ChIP-seq
x		Flow cytometry
x		MRI-based neuroimaging

Eukaryotic cell lines

Policy information about cell lines

Cell line source(s)	WT (C57BL/6) or Polm-/- Poll-/- double knock out murine fibroblast (MEF) cells (generously provided by Dr. L. Blanco) were derived from E14.5d embryos and immortalized by the introduction of SV40 large T-antigen.
Authentication	The Poll−/−Polm−/− cell line genotype was initially confirmed using PCR and Western blot analysis (citation: https://pubmed.ncbi.nlm.nih.gov/26240371/). As described in this paper, this cell line was shown to be incapable of accurate repair of both a Pol mu cognate substrate (3' G noncomplementary overhang) and a Pol lambda cognate substrate (3'GCAG complementary overhang). This cell line is routinely validated using these functional assays.
Mycoplasma contamination	These lines were confirmed by qPCR to be free of mycoplasma contamination.
Commonly misidentified lines (See ICLAC register)	No commonly misidentified cell lines were used in this study.
